# Spatial Dissection of Invasive Front from Tumor Mass Enables Discovery of Novel microRNA Drivers of Glioblastoma Invasion

**DOI:** 10.1002/advs.202101923

**Published:** 2021-11-01

**Authors:** Yulun Huang, Lin Qi, Mari Kogiso, Yuchen Du, Frank K. Braun, Huiyuan Zhang, L. Frank Huang, Sophie Xiao, Wan‐Yee Teo, Holly Lindsay, Sibo Zhao, Patricia Baxter, Jack M. F. Su, Adekunle Adesina, Jianhua Yang, Sebastian Brabetz, Marcel Kool, Stefan M. Pfister, Murali Chintagumpala, Laszlo Perlaky, Zhong Wang, Youxin Zhou, Tsz‐Kwong Man, Xiao‐Nan Li

**Affiliations:** ^1^ Department of Neurosurgery Dushu Lake Hospital Soochow University Suzhou 205124 China; ^2^ Department of Neurosurgery and Brain and Nerve Research Laboratory the First Affiliated Hospital Soochow University Suzhou 215007 China; ^3^ Texas Children's Cancer Center Texas Children's Hospital Baylor College of Medicine Houston TX 77030 USA; ^4^ Program of Precision Medicine PDOX Modeling of Pediatric Tumors Ann & Robert H. Lurie Children's Hospital of Chicago Department of Pediatrics Northwestern University Feinberg School of Medicine Chicago IL 60611 USA; ^5^ Department of Pharmacology School of Medicine Sun Yat‐Sen University Shenzhen 518107 China; ^6^ Department of Systems Medicine and Bioegineering Houston Methodist Hospital Research Institute and Cancer Center Weill Cornell Medicine Houston TX 77030 USA; ^7^ Division of Experimental Hematology and Cancer Biology Brain Tumor Center Cincinnati Children’s Hospital Medical Center Department of Pediatrics University of Cincinnati College of Medicine Cincinnati United States 45229 United States; ^8^ Humphrey Oei Institute of Cancer Research National Cancer Center Singapore Singapore 169610 Singapore; ^9^ Department of Pathology Texas Children's Hospital Baylor College of Medicine Houston TX 77030 USA; ^10^ Hopp Children's Cancer Center (KiTZ) Heidelberg 69120 Germany; ^11^ Division of Pediatric Neuro‐oncology German Cancer Research Center (DKFZ) and German Cancer Consortium (DKTK) Heidelberg 69120 Germany; ^12^ Department of Pediatric Hematology and Oncology Heidelberg University Hospital Heidelberg 69120 Germany

**Keywords:** 4‐aminopyridine, glioblastoma, KCNA1, miRNA, patient derived orthotopic xenograft

## Abstract

Diffuse invasion is the primary cause of treatment failure of glioblastoma (GBM). Previous studies on GBM invasion have long been forced to use the resected tumor mass cells. Here, a strategy to reliably isolate matching pairs of invasive (GBM*^INV^*) and tumor core (GBM*^TC^*) cells from the brains of 6 highly invasive patient‐derived orthotopic models is described. Direct comparison of these GBM*^INV^* and GBM*^TC^* cells reveals a significantly elevated invasion capacity in GBM*^INV^* cells, detects 23/768 miRNAs over‐expressed in the GBM*^INV^* cells (miRNA*^INV^*) and 22/768 in the GBM*^TC^* cells (miRNA*^TC^*), respectively. Silencing the top 3 miRNAs*^INV^* (miR‐126, miR‐369‐5p, miR‐487b) successfully blocks invasion of GBM*^INV^* cells in vitro and in mouse brains. Integrated analysis with mRNA expression identifies miRNA*^INV^* target genes and discovers *KCNA1* as the sole common computational target gene of which 3 inhibitors significantly suppress invasion in vitro. Furthermore, in vivo treatment with 4‐aminopyridine (4‐AP) effectively eliminates GBM invasion and significantly prolongs animal survival times (*P* = 0.035). The results highlight the power of spatial dissection of functionally accurate GBM*^INV^* and GBM*^TC^* cells in identifying novel drivers of GBM invasion and provide strong rationale to support the use of biologically accurate starting materials in understanding cancer invasion and metastasis.

## Graphical abstract

Using 6 highly invasive glioblastoma (GBM) PDOX models, this study isolates matching pairs of invasive (GBM*^INV^*) and tumor core (GBM*^TC^*) cells, demonstrates an elevated invasion capacity in GBM*^INV^* cells, identifies a novel miRNA signature (miRNA*^INV^*) of GBM invasion, and discovers a commonly shared gene, KCNA1, from miRNA*^INV^* regulated genes that can be pharmacologically inhibited to block GBM invasion in vivo.

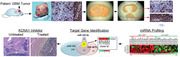

## 1 Introduction

Glioblastoma multiforme (GBM) is the most malignant brain tumor in children and adults. Despite multimodal therapies and significant advances in the understanding of tumor biology^[^ ^1^ ^]^ and molecular subgroups,^[^ ^2^, ^3^, ^4^ ^]^ the prognosis of patients with GBM remains extremely poor,^[^ ^5^ ^]^ with 5‐year survival rates between 5% and 15% in children^[^ ^6^ ^]^ and a 1‐year survival rate of ≈10% in adults.^[^ ^7^ ^]^ Diffuse infiltration of tumor cells into surrounding normal brain tissue, a hallmark of GBM growth, is the primary cause of tumor recurrence and treatment failure.^[^ ^8^ ^]^ While major advances have been made in understanding the biology of GBM by studying cells from the tumor core, little is known about the invasive GBM cells (GBM*^INV^*) that migrate deep into surrounding normal brain tissues despite the findings of “go or grow” mechanism^[^ ^9^, ^10^ ^]^ and epithelial to mesenchymal transition.^[^ ^11^, ^12^ ^]^ This is because these GBM*^INV^* cells are not amenable to surgical removal for study, as aggressive surgical resection of normal tissue carries the risk of serious and permanent neurological deficits.^[^ ^6^, ^13^ ^]^ Additionally, although it has been suggested that the blood‐brain barrier (BBB) could be compromised and consequently “leaky” in the GBM tumor core (GBM*^TC^*), GBM*^INV^* cells are frequently protected by an intact and functional BBB, making them even less vulnerable to chemotherapeutic agents than GBM*^TC^* cells.

Most past and current studies on GBM invasion were and are still forced to utilize tumor tissues resected from the primary tumor mass for biologic analyses. Although these GBM*^TC^* cells may be capable of invasive growth, they are not actively invading at the time of harvest. Since tumor invasion is a complex biological process involving functional modifications and dynamic interactions between tumor cells and the microenvironment, there remains an urgent need for biologically‐ and functionally‐accurate GBM*^INV^* cells to identify key genetic drivers of GBM invasion.

To overcome this barrier, we developed a panel of patient‐derived orthotopic xenograft (PDOX or orthotopic PDX) mouse models through direct implantation of pediatric GBM (pGBM) surgical specimens into matched locations in the brains of SCID mice. Detailed characterization showed that these PDOX tumors replicate the histopathological features of pGBM, maintained key genetic abnormalities of the original patient tumors, and importantly, remained highly invasive.^[^ ^14^, ^15^, ^16^ ^]^ These transplantable PDOX models thus provide a reliable resource for isolating paired and functionally accurate GBM*^INV^* cells from normal mouse brain tissues/parenchyma and GBM*^TC^* cells from the tumor core for biological studies of pGBM invasion. The inclusion of different molecular subtypes of pGBMs should further facilitate the discovery of commonly shared or subtype‐specific biological changes.

Accumulating data suggest that microRNAs (miRNA), noncoding RNAs of ≈20–23 bps,^[^ ^17^, ^18^ ^]^ play a key role in GBM invasion.^[^ ^19^ ^]^ miRNAs often bind to target mRNAs through partial complementary pairing and either suppress mRNA translation or reduce mRNA stability. They are shown to regulate multiple cellular processes including cell division, differentiation, and death.^[^ ^20^ ^]^ While the study of miRNAs in pGBM is still in its infancy, over‐expression of miRNAs, including miR‐34a, ‐124, ‐128, ‐137, and ‐145, has been detected in adult GBM and shown to suppress self‐renewal, inhibit tumorigenesis,^[^ ^21^ ^]^ trigger cell cycle arrest,^[^ ^22^ ^]^ or apoptosis,^[^ ^23^, ^24^ ^]^ and promote invasion in vitro in cultured GBM cells and in vivo in subcutaneous xenografts.^[^ ^19^, ^25^ ^]^

In this study, we utilized 6 PDOX mouse models of pGBM of different subtypes to harvest functionally accurate, paired GBM*^INV^* cells (that have invaded into the surrounding normal mouse brain tissue) and GBM*^TC^* cells (from the primary tumor mass) to examine their functional differences in migrating into normal brains, followed by the analysis of differentially expressed miRNAs via global microRNA profiling, validation of the functional roles of the candidate driver miRNAs both in vitro and in vivo in mouse brains using purified GBM*^INV^* cells. We subsequently examined genes that mediate the driver miRNA‐induced pGBM invasion, and finally identified a novel candidate driver gene *KCNA1* and demonstrated the therapeutic efficacy of pharmacological targeting of this invasion‐driver gene in blocking pGBM invasion and prolonging PDOX survival times.

## 2 Results

### 2.1 Patient‐Derived Orthotopic Xenograft Tumors Replicate the Highly Invasive Phenotype of Pediatric Glioblastoma In Vivo

**Figure 1:**
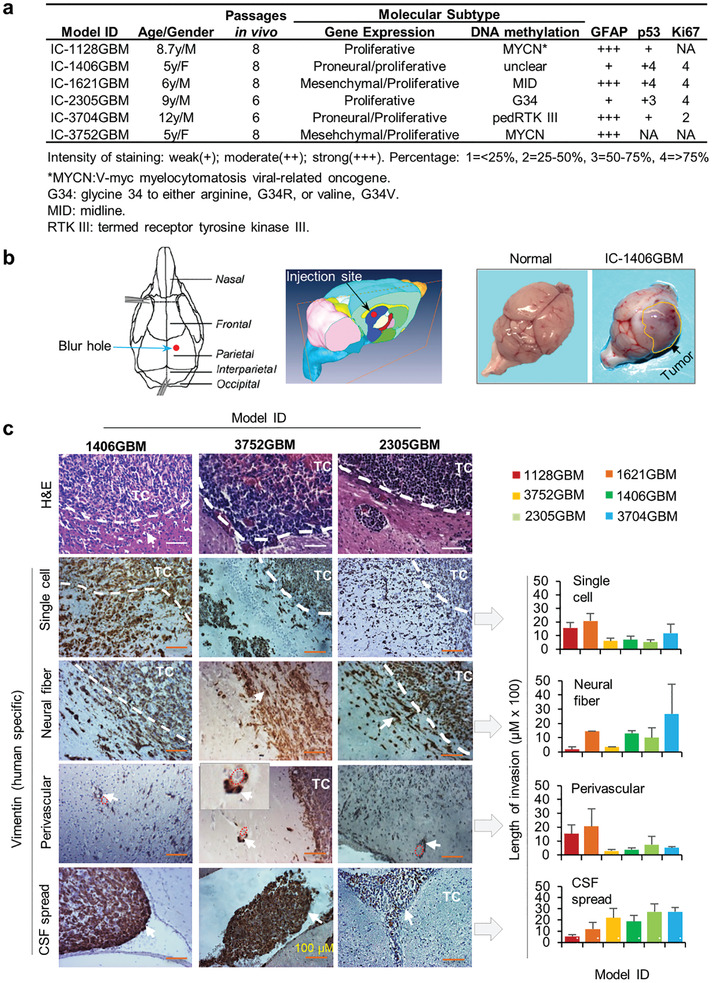
PDOX pGBM tumors were highly invasive in vivo. a) Clinical, pathological, and molecular subtype information of the six pGBM tumors. b) Orthotopic pGBM tumor implantation strategy and representative gross appearance of pGBM PDOX models. Tumor cells (1 × 10^5^ in 2 µL) from six pGBMs were directly implanted into right cerebra of NOD/SCID mm mice (1.5 mm anterior and 3 mm deep) (left panel). The animals were monitored daily until they developed neurological deficits or became moribund, at which time they were euthanized. Formation of PDOX tumors can frequently be observed (arrow, right panel). c) Representative images showing the modes of intra‐cerebral invasion in PDOX models (left panel) and the quantitative analysis of migration distances of all six models (right panel). In additional to H&E staining, human pGBM xenograft cells were positively identified through IHC staining using human‐specific antibodies against vimentin (arrow). In all six models, invasion through single cells, along blood vessels (perivascular invasion), along neural fibers, and spread through cerebral spinal fluid (CSF) were observed. The distances between the invasive front and the border line of tumor core (white dotted line) were measured and graphed. Scale bar = 100 µM. *n* = 3 in each condition. Data are shown as mean ± SD.

Diffuse infiltration of tumor cells into surrounding normal brain tissue is one of the hallmarks of GBM. To confirm that our pGBM PDOX models replicated this important biological feature, we performed a systematic analysis of the invasive capacity and mode of tumor cell migration of pGBM cells in six PDOX models that had been sub‐transplanted in vivo in mouse brains for 6–8 generations as described previously (**Figure** **1**a,b).^[^ ^15^, ^26^ ^]^ These models were subgrouped as proliferative, proneural, and mesenchymal through gene expression analysis (Figure 1a),^[^ ^2^ ^]^ and MYCN, MID, G34, and pedRTKIII subtypes with DNA methylation profiling(Figure 1a).^[^ ^27^, ^28^ ^]^ When the tumor‐bearing mice became moribund, whole mouse brains were harvested, paraffin embedded, and serially sectioned (>160 sections/mouse brain). Standard H&E staining consistently revealed a large tumor mass surrounded by a characteristic “ragged edge” (Figure 1c and Figure S1, Supporting Information). To positively identify single and/or small clusters of invasive pGBM cells, we performed immunohistochemical (IHC) staining using human‐specific antibodies against vimentin (VIM)^[^ ^15^, ^29^, ^30^ ^]^ and used a straight line reticle (eyepiece micrometer) to measure the distances of migration under a Nikon 3 head teaching microscope from which 2–3 investigators can examine the same fields of the same slides at the same time. In tumors with short invasion that can be captured in one image (from 4× to 20×), ImageJ was applied to digitally measure the distances. The border line between tumor core and invasion (or the edge of tumor core) was defined as the front of tumor core where tumor cells were lined up facing normal brain tissues; while the leading front of invasion was determined by the tumor cells that migrated the farthest (or deepest) into the normal brain. A line drawing from the leading front invasive cells perpendicular to the tumor core edge was used to measure the distances (Figure 1c). For each PDOX models, at least three mouse brains each with >3 slides of the largest cross sections of tumor mass were included. Despite the different molecular subtypes, diffuse invasion was detected in all six models (Figure 1c and Figure S1, Supporting Information). Most of the leading invasive edge was composed of single tumor cells followed by increasingly larger micro‐tumors closer to the tumor core, covering a distance ranging from 525 to 2083 µM (mean 1109 ± 375.2 µm). Migration along blood vessels, that is, perivascular invasion,^[^ ^31^ ^]^ was detected in all 6 models, ranging from 289.2 to 2058.3 µm (914.4 ± 466.7 µm); while invasion along neural fibers ranged from 170 to 2666.7 µm (1158 ± 524 um). Seeding into the cerebral spinal fluid (CSF) was observed in some sections; the distances varied from 525 to 2750 µm (1881.3 ± 541.1 µm) (Figure 1c). Together, these data demonstrated maintenance of the invasive pGBM phenotype and all 3 routes of GBM tumor migration in vivo in our PDOX models, with single cell migration as the predominating mode.

### 2.2 Spatial Dissection to Isolate Matching Pairs of Invasive (GBM*^INV^*) and Tumor Core (GBM*^TC^*) Cells

**Figure 2:**
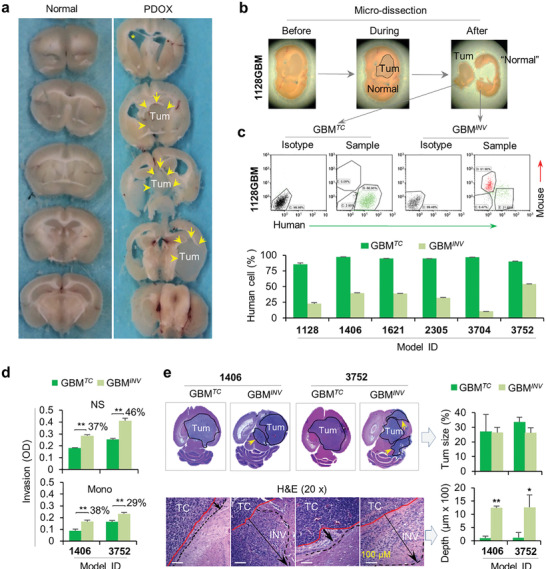
GBM*^INV^* cells possess stronger invasive capacity than their matching GBM*^TC^* cells. a) Slicing of fresh whole mouse brain to facilitate easy identification of tumor core. Whole mouse brains were placed on a mouse brain matrix and sliced at 1 mm thickness into 10–12 slices. b) Separation of tumor core (tumor) from “normal” mouse brains under stereotactic microscope. The border between tumor and normal (circle) was identified following general guidelines of human brain tumor resection during surgery.^[^ ^32^, ^33^, ^34^ ^]^ “Normal” mouse brain tissues (containing GBM*^INV^* cells) and tumor mass (containing GBM*^TC^* cells) were placed in cold (4 °C) growth medium in separate Petri dishes and dissociated into single cell suspension using Gentle Dissociator (Miltenyi). c) Purification of GBM*^INV^* and GBM*^TC^* cells through FACS. Cell suspensions were incubated with FITC‐conjugated monoclonal antibodies against human HLA‐ABC and APC‐conjugated monoclonal antibodies against mouse major histocompatibility antigen by FACS. The mouse cells (APC‐positive and FITC‐negative) were gated out together with the dead cells (propidium iodine high). Data are shown as mean ± SD. d) In vitro assay showing significantly increased invasive capacity of GBM*^INV^* cells than the matching GBM*^TC^* cells in two models under two different growth conditions. The purified GBM*^INV^* and GBM*^TC^* cells from IC‐1406GBM (1406) and IC‐3752GBM (3752) were cultured as neurosphere (NS) in serum‐free media supplemented with EGF and bFGF and monolayer (Mono) cells in traditional FBS‐based medium. The invasive capacity of GBM*^TC^* and GBM*^INV^* was examined in triplicates by CytoSelect 96‐Well Cell invasion assay (*P < 0.05) (Data are shown as mean ± SD). e) In vivo validation of higher invasive capacity of GBM*^INV^* cells than that of the matching GBM*^TC^* cells. Purified GBM*^INV^* and GBM*^TC^* cells from IC‐1406GBM (1406) and IC‐3752GBM (3752) models were implanted separately into the brains of SCID mice. The animals were euthanized when they develop signs of neurologic deficits or become moribund. Paraffin sections were stained with H&E and the distances from the “border” of tumor core (red line) to the far front of the invasive edge (black line) were measured (arrow). Note the formation of invasive satellite tumors in mouse brains implanted with GBM*^INV^* cells (arrow in the upper panel). Tumor sizes and depths of pGBM invasion were quantitated by ImageJ (**P < 0.01, *P < 0.05) (*n* = 3 per group. Data are shown as mean ± SD).

To isolate GBM*^INV^* and GBM*^TC^* cells, freshly harvested whole mouse brains were sectioned into 1 mm slices to enable gross identification of the primary tumor mass (**Figure** **2**a) and to facilitate microscopic dissection of the tumor core (from which GBM*^TC^* cells were collected) from “normal” mouse brain tissue (from which GBM*^INV^* cells were collected) (Figure 2b).^[^ ^32^, ^33^, ^34^ ^]^ To purify human GBM*^INV^* and GBM*^TC^* cells, we utilized FITC‐conjugated human HLA‐ABC antibodies and a cocktail of APC‐conjugated antibodies specific to mouse CD24, CD90, CD117, CD133 and performed florescence activated cell sorting (FACS). In the invasive front (from “normal” mouse brain tissues), human HLA‐ABC^+^ cells (GBM*^INV^*) ranged from 10.6% to 54.4% (33.0 ± 0.68%) of the viable cells; in the tumor core, human GBM*^TC^* cell proportions ranged from 85.9% to 97.2% (93.1 ± 0.58%) (Figure 2c and Figure S2, Supporting Information). These data provided a quantitative estimate of the diffusive invasion of pGBM in mouse brains, using a novel strategy to harvest matched pairs of functionally distinct GBM*^INV^* and GBM*^TC^* cells for biological studies.

### 2.3 GBM*^INV^* Cells Possess Significantly Stronger Invasive Capacity both In Vitro and In Vivo

To test our hypothesis that GBM*^INV^* cells possess stronger migratory capacity than those in the GBM*^TC^* cells, we first compared their invasive capability in vitro with a standard invasion assay. Since monolayer tumor cells maintained in traditional fetal bovine serum (FBS)‐based media do not share biological features with neurospheres propagated in serum‐free media (supplemented with EGF and *β*FGF), which favors the growth of cancer stem cells,^[^ ^15^, ^30^, ^35^, ^36^ ^]^ we incubated pGBM cells in both types of growth media to have a better coverage of cell subpopulations and to understand the differences between the monolayer and the neurosphere cells. GBM*^INV^* cells from two pGBM models (IC‐1406GBM and IC‐3752GBM) exhibited a 29–46% increase in invasion compared to the matching GBM*^TC^* cells (*P* <0.05), and 3D neurosphere cells were significantly more invasive (37–46% higher) than the monolayer cells (Figure 2d). Since neurospheres grew in suspension, the scratch assay that measures the cell motility was not performed.

To further validate these findings in vivo, we directly implanted freshly purified GBM*^INV^* and GBM*^TC^* cells from these two pGBM models into the brains of SCID mice and examined their invasive capacity on paraffin sections via H&E and IHC staining. As anticipated, invasion into surrounding normal brain was observed in the xenografts derived from GBM*^TC^* cells, confirming the maintenance of invasive capacity of a fraction of GBM*^TC^* cells even though they were not “invading” at the time of harvesting. In xenografts derived from GBM*^INV^* cells, however, the depth of invasion was significantly longer compared to GBM*^TC^* cells (1246 vs 105.1 µm in IC‐1406GBM and 1266.3 vs 114 µm in IC‐3752GBM) (Figure 2e). While GBM*^TC^* cells formed large intra‐cerebral tumor masses, it was the GBM*^INV^* cells that developed invasive satellite tumors (Figure 2e). These in vitro and in vivo data demonstrated the functional differences between GBM*^INV^* and GBM*^TC^* cells, thereby highlighting the importance of using functionally accurate GBM*^INV^* cells in understanding GBM invasion.

### 2.4 Novel miRNA Drivers of Pediatric Glioblastoma Invasion

**Figure 3:**
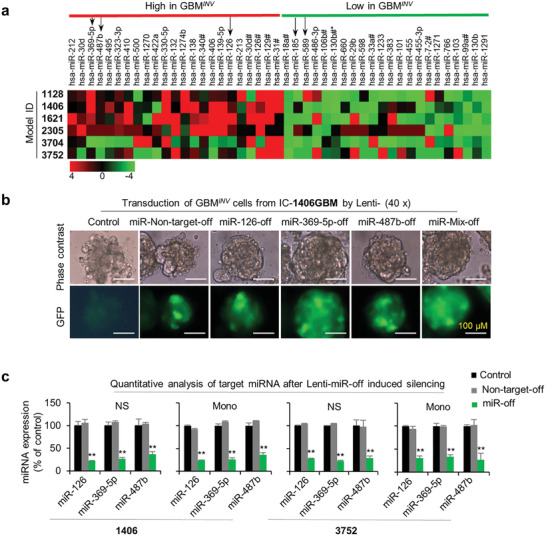
Differentially expressed miRNAs between GBM*^INV^* and GBM*^TC^* cells. a) Hierarchical clustering of over‐expressed and downregulated miRNA in GBM*^INV^* cells. miR‐126, miR‐487b, and miR‐369‐5p were selected for functional study from the 23 upregulated miRNAs, and miR‐185 and miR‐589 were selected from the 22 downregulated miRNAs in least 4 of the 6 pGBM models (*P* < 0.05). b) Representative images showing successful infection of pGBM cells by Lentivirus‐miR‐GFP. The purified GBM*^INV^* cells from IC‐1406GBM were grown as neurospheres and infected with lentivirus‐miRNA‐off (miR487b‐off, miR‐126‐off, miR‐369‐5p‐off, and mix‐off) for 72 h (MOI 1:1) and examined for the expression of GFP. Non‐infected cells were included control. c) Confirmation of lentivirus‐mediated miRNA knock‐down of miRNA*^INV^* using RT‐qPCR. Both neurosphere (NS) and monolayer (Mono) cells derived from GBM*^INV^* cells of IC‐1406GBM (1406) and IC‐3752GBM (3752) were tested. (**P < 0.05) (Data are shown as mean ± SD).

MiRNAs have been implicated in driving biological processes of human cancers^[^ ^37^ ^]^ including tumor invasion and metastasis.^[^ ^38^, ^39^ ^]^ To test our hypothesis that GBM*^INV^* cells depend on a unique set of miRNAs for invading into normal brain tissue, we compared the miRNA expression profiles of matched GBM*^INV^* and GBM*^TC^* cells derived from the 6 PDOX pGBM models using TaqMan MicroRNA array. In addition to four control assays provided by the vendor, we included normal human cerebral tissue obtained from warm autopsy (≈4 h postmortem) as a reference. Differences in miRNA expression between GBM*^INV^* and GBM*^TC^* cells were directly compared and the fold changes calculated by 2^−ΔCT^. Of the 768 miRNAs detected, 23 were significantly upregulated (>twofold) in the GBM*^INV^* cells (hereafter designated as miRNA*^INV^*), and 22 miRNAs upregulated in GBM*^TC^* cells (hereafter referred as miRNA*^TC^*) in at least 4 of the 6 pGBM models (**Figure** **3**a and Table S1, Supporting Information).

### 2.5 Silencing miRNA*^INV^* in GBM*^INV^* Cells Suppresses Invasion In Vitro

To examine the functional role of the upregulated miRNAs in the GBM*^INV^* cells (i.e., miRNA*^INV^*) we selected two newly discovered miRNAs that exhibited high fold changes and high frequency, that is, miR‐369‐5p with >3.11‐folds in 5/6 models and miR‐487‐5p with > 3.3‐fold in 4/6 models, as well as, one miRNA (miR‐126, >2.7‐fold in 5/6 models) that was reported to be involved in tumor invasion.^[^ ^40^, ^41^ ^]^ We examined the effects of loss‐of‐function and gain‐of‐function of these 3 microRNAs through lentivirus‐mediated transduction assays. Successful transduction was confirmed with Lenti‐GFP (Figure 3b) and the efficient knock‐down of target miRNA*^INV^* (>70%) with RT‐qPCR (Figure 3c).

**Figure 4:**
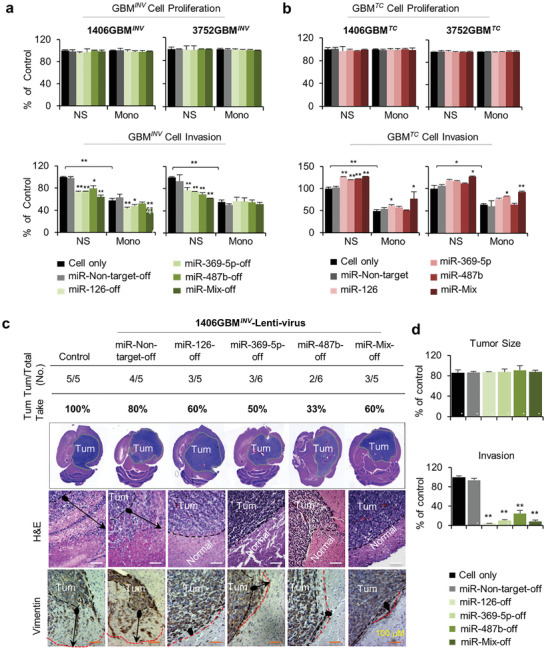
Functional validation of miRNA*^INV^* in GBM*^INV^* cell invasion both in vitro and *vivo*. a) In vitro loss‐of‐function assay showing the suppression of GBM*^INV^* cell invasion by lentivirus mediated silencing of miRNAs*^INV^*. The puromycin‐selected and FACS‐purified GFP^+^ GBM*^INV^* cells from IC‐1406GBM (1406GBM ***^IN^*** *^V^*) and IC‐3752GBM (3752GBM ***^INV^***) were examined both as neurospheres (NS) and monolayer cells (Mono). Data were normalized to the cell only group of neurospheres and presented as % of control. While cell proliferation was not affected (*P* > 0.05) (top panel), silencing of miR‐126, ‐369‐5p, ‐487b alone, and in combination (miR‐mix‐off) with Lentivirus‐miRNA‐off led to significant suppression of cell invasion (lower panel, *P* < 0.05) as examined by CytoSelect 96‐Well Cell Invasion Assay. (**P < 0.01, *P < 0.05 compared to the control group, *n* = 3. Data are shown as mean ± SD). b) In vitro gain‐of‐function assay showing the activation of GBM*^TC^* cells following lentivirus mediated transduction of miRNA*^INV^*. GBM*^TC^* cells (that were not actively invading) were transduced with Lentivirus‐miRNA to increase the expression of the miRNA*^INV^*. Cell proliferation was not affected (upper panel), but cell invasion was increased, particularly in neurosphere (NS) cells from IC‐1406GBM (1406GBM ***^TC^***) and IC‐3752GBM (3752GBM ***^TC^***) (**P < 0.01, *P < 0.05 compared to the control group, *n* = 3. Data are shown as mean ± SD). c) In vivo confirmation of suppressed GBM*^INV^* cell invasion following the silencing of miRNA*^INV^* s. GBM*^INV^* cells from IC‐1406GBM (1406GBM ***^IN^*** *^V^*) were transduced with Lentivirus‐miRNA‐off followed by puromycin‐selection. The FACS‐purified GFP^+^ GBM*^INV^* cells were implanted into the brains of NOD/SCID mice (1 × 10^5^ cells per mouse brain) and monitored for signs of neurological deficits or moribund when the animals were euthanized. 1406GBM*^INV^* cells were identified through H&E staining of whole mouse brains and IHC staining of paraffin‐embedded sections using human‐specific antibodies against VIM. d) The slides with the largest cross section of intra‐cerebral xenografts were examined to quantitatively evaluate the tumor size and the distances (arrow)between the invasive front (red line) and tumor core “board line” (black dotted line) were measured by ImageJ (*P < 0.05). Scale bars represent 100 µM. Data are shown as mean ± SD.

For the loss‐of‐function analysis of miRNA*^INV^*, the invasive capacity of puromycin‐selected and GFP^+^ GBM*^INV^* cells from two highly invasive PDOX models, IC‐1406GBM and IC‐3752GBM, was examined using CytoSelect 96‐Well Cell Invasion Assay in quadruplicates. As shown in **Figure** **4**a, silencing miR‐126, ‐369‐5p, and ‐487b with Lenti‐miRNA‐126‐off, ‐369‐5p‐off, and ‐487b‐off, (MOI = 1:1 for 72 h) did not affect cell proliferation in either the GBM*^INV^* monolayer or neurosphere cultures but induced significant suppression of invasion in GBM*^INV^* cells grown as neurospheres (*P* < 0.05 compared to the untreated and the GBM*^INV^* cells transduced with Lenti‐non‐target‐off, *n* = 3) from both pGBM models. In the monolayer cells, only invasion of IC‐1406GBM*^INV^* cells was inhibited (Figure 4a and Figure S3a, Supporting Information). These data indicated the selectivity of the miRNA*^INV^* function in cell invasion, particularly in the 3D neurospheres that exhibited increased invasive capacity (in Section 2.3), and support their role as candidate miRNA drivers of pGBM invasion.

To determine if gain‐of‐function of miRNA*^INV^* promotes invasion in GBM*^TC^* cells, which have lower levels of these 3 miRNAs, we transduced the non‐invading tumor core cells IC‐1406GBM*^TC^* and IC‐3752GBM*^TC^* cells with Lenti‐miRNA‐126, ‐487b, and ‐369‐5p in quadruplicates. The increased expression of these miRNA*^INV^* (alone or in combination) did not alter cell proliferation, similar to GBM*^INV^* cells (Figure 4b and Figure S3b, Supporting Information) but resulted in significantly elevated invasion in 3D neurospheres of IC‐1406GBM*^TC^* and in monolayers of IC‐3752GBM*^TC^*. In monolayers of IC‐1406GBM*^TC^* and neurospheres of IC‐3752GBM*^TC^*, overexpression of a single miRNA*^INV^* failed to promote invasion; however, simultaneous overexpression of all 3 miRNA*^INV^* caused a significant increase in invasion (Figure 4b and Figure S3b, Supporting Information). These data indicated collective/cooperative activities of these miRNA*^INV^* in promoting pGBM invasion and suggested a complex nature of the underlying biology of GBM invasion.

### 2.6 Silencing miRNA*^INV^* Significantly Suppresses Pediatric Glioblastoma Invasion in Mouse Brains

GBM invasion is an active process involving dynamic interactions between tumor cells and their microenvironment. To validate the functional roles of miRNA*^INV^* in vivo in a microenvironment similar to human brain tissue, FACS‐purified IC‐1406GBM*^INV^* cells were transduced with Lenti‐mir‐126‐off, ‐369‐5p‐off, and ‐487b‐off (MOI 1:1) to silence the 3 miRNA*^INV^* and subsequently implanted into the brains of NOD/SCID mice (1 × 10^5^ cells/mouse brain, *n* = 5 per group). Compared with the 100% (5/5) tumor uptake rate seen with untreated IC‐1406GBM*^INV^* cells and 80% (4/5) in the non‐target lentivirus‐transduced group, the tumor take rates were reduced to 60% (3/5) after implantation of cells with Lenti‐miRNA‐126‐off, 50% (3/6) with Lenti‐miRNA‐369‐5p‐off, 33% (2 of 6) with Lenti‐miRNA‐487b‐off, and 60% (3/5) with a combination of all 3 Lenti‐miRNA‐off (Figure 4c). Animal survival times were not significantly different among the tumor‐bearing mice (Figure S5, Supporting Information).

We next examined whether silencing the 3 miRNA*^INV^* blocked GBM*^INV^* invasion in vivo in mouse brains. Except for the miR‐487b‐off group, in which 2 mouse brains were analyzed, there were 3 mouse brains in all the remaining groups (control, non‐target, miR126‐off, miR‐369‐5p‐off, and miR‐combination‐off). To quantitatively evaluate the invasive potential, slides with the largest cross‐section of intra‐cerebral xenografts were examined (Figure 4c). The non‐target control (miR‐non‐target‐off) and untreated IC‐1406GBM*^INV^* exhibited similar invasive capacity. Lentiviral‐mediated silencing of miR‐126, ‐369‐5p, and ‐487b caused significant reduction of invasion depth, ranging from >75% by miR‐487b‐off to ≈90% by miR‐369‐5p‐off and >95% by miR‐126‐off (Figure 4d). When the tumor sizes were compared, the differences among the six groups were not significantly different (Figure 4d) and IHC examination of stem cell (Nestin), neural (MAP2), glial (GFAP), cell proliferation (Ki67), and mitochondrial markers failed to reveal major differences as well (Figure S6, Supporting Information). Altogether, silencing the miRNA*^INV^* (miRNA‐487b, ‐369‐5p, and ‐126) in GBM*^INV^* cells blocked pGBM invasion and reduced tumorigenicity in vivo, supporting a critical role of these miRNA*^INV^* in maintaining the invasive phenotype of pGBM cells.

### 2.7 miRNAs*^INV^* Targeted a Set of Shared Genes and Signaling Pathways

miRNA‐mediated gene regulation is very complex.^[^ ^42^ ^]^ The activity of a given miRNA on a transcript may result in target mRNA degradation, blockage of translation, or increased mRNA expression.^[^ ^18^ ^]^ Further, a single miRNA can target multiple mRNAs to coordinately regulate their expression; in contrast, multiple miRNAs can target a single mRNA. To identify the mRNA targets of the 3 miRNA*^INV^*, we 1) searched TargetScan for an updated list of target genes of the 3 miRNA*^INV^* (miR‐126, ‐487b, and ‐369‐5p), 2) performed global gene expression profiling in the same six pairs of GBM*^INV^* and GBM*^TC^* cells using normal childhood cerebral RNA as control, and 3) generated a list of differentially expressed genes of the 3 miRNA*^INV^* between GBM*^INV^* and GBM*^TC^*, (fold difference >1.5 or <0.5, *P_INV/TC_* < 0.05). The *P*‐values of differentially expressed genes between GBM*^INV^* and normal human cerebral tissues (*P_INV/Normal_*) and between GBM*^TC^* and normal tissues (*P_TC/Normal_*) were also calculated.

**Figure 5:**
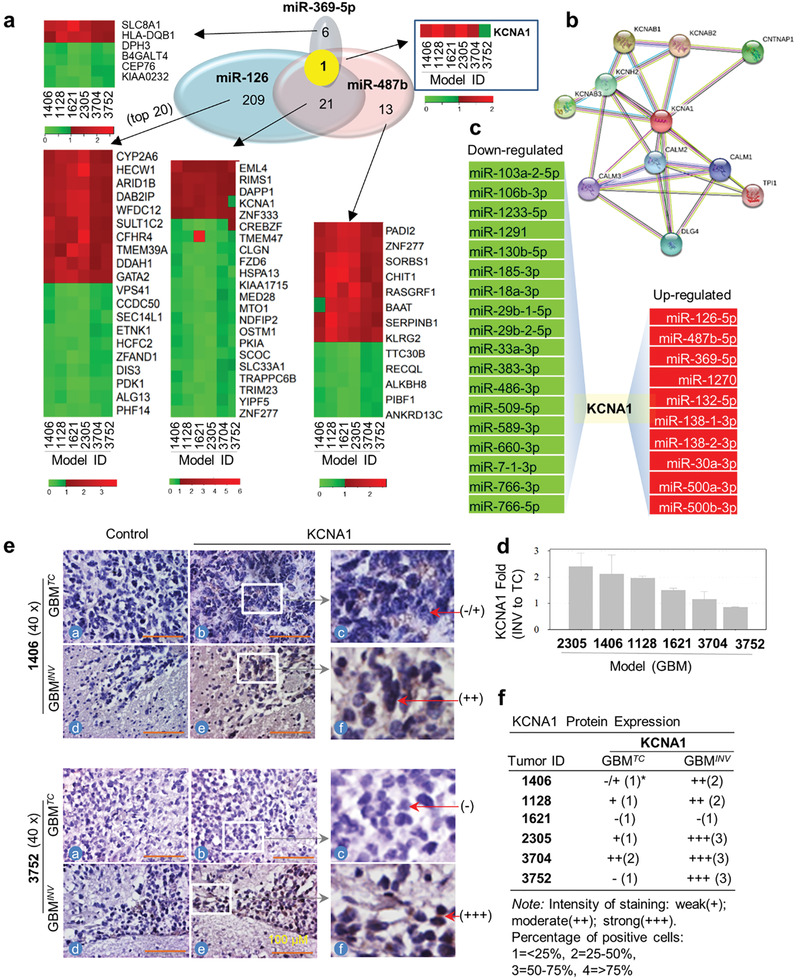
*KCNA1* is a common target gene of miRNA*^INV^*. a) Integrated analysis of mRNA profiling with the 3 miRNA*^INV^* via TargetScan identified the private and shared target genes of miR‐126, ‐487b, and ‐369‐5p. *KCNA1* is the only gene commonly targeted by all 3 miRNA*^INV^*.b) Protein‐protein interaction network analysis with STRING identified major binding partners of *KCNA1*. c) List of down‐ or upregulated miRNAs in the GBM*^INV^* cells that also target *KCNA1*. d) Over‐expression of *KCNA1* mRNA in the GBM*^INV^* cells compared with the matching GBM*^TC^* cells in 4/6 pGBM models examined. Data are shown as mean ± SD. e,f) Elevated expression of KCNA1 protein in vivo in the invasive front (GBM*^INV^*) as compared with the tumor core (GBM*^TC^*) cells as analyzed with IHC in the six pGBM PDOX models. Slides incubated without the primary antibodies were uses as control.

For miR‐126, a total of 231 target genes were differentially expressed, including 126 downregulated (<0.5‐fold) and 105 upregulated genes (>1.5‐fold) in GBM*^INV^* compared with GBM*^TC^* cells (**Figure** **5**a and Table S2, Supporting Information). For miRNA‐487b, there were 37 target genes (23 downregulated and 14 upregulated) (Figure 5a and Table S3, Supporting Information), 22 (62.8%) of which were shared targets of miR‐126 (Figure 5A). For miR‐369‐5p, which has not been associated with any human disease, only seven target genes were found (Figure 5a and Table S4, Supporting Information). The levels of most of the genes identified in the tumor core (GBM*^TC^*) were not significantly different from those in normal human cerebral tissues, including 203/247 (81.8%) genes targeted by miR‐126, 33/35 (94.2%) by miR‐487b, and 6/7 (85.7%) by miR‐369‐5p (Tables S2–S4, Supporting Information). Therefore, these genes could have been missed if only GBM*^TC^* cells were utilized to compare with normal tissues.

Pathway enrichment analysis of the differentially expressed target genes was performed through Ingenuity with Fisher's exact test. When examined individually, each of the 3 miRNA*^INV^* affected a distinct collection of pathways. For example, the top canonical pathways affected by miR‐126 were dTMP novo biosynthesis, cardiomyocyte differentiation, cardiac‐adrenergic, cAMP‐mediated signaling, and ERK/MAPK signaling. The top miR‐487b‐affected pathways were protein citrullination, neutral pathway, pyrimidine ribonucleotide interconversion, and the Wnt/Ca+ pathway. Although miR‐369‐5p only targeted nine genes, the top affected pathways were B‐cell development, antigen presentation, and the autoimmune thyroid disease pathway (Figure S7a, Supporting Information). When analyzed for the shared pathways, all 3 miRNA*^INV^* regulated cell‐to‐cell signaling and cell interaction (Figure S7b, Supporting Information), while organismal injury and abnormalities, and nervous system development and function were affected by 2 of the 3 miRNA*^INV^* (Figure S7b, Supporting Information). Collectively, the 3 miRNA*^INV^* modulated ERK/MAPK and cAMP‐mediated signaling (in canonical pathways) (Figures S7a, S8b–S11b, Supporting Information), cancer, organismal injury (in disease) (Figure S7b, Supporting Information), protein synthesis, cellular development, cell death and survival, cell‐to‐cell signaling, and interaction (in molecular functions) (Figures S7b, S8b–S11b, Supporting Information), and embryonic, organ, organismal, reproductive system, and tissue development (in physiological systems involved in cancer invasion and metastasis) (Figures S8b–S11b, Supporting Information).

To further deduce the protein networks, we used STRING,^[^ ^43^ ^]^ a database tool for predicting protein‐protein interactions directly and indirectly for analyzing miRNA*^INV^* targeted networks. In miR‐126 targeted genes, 2 major networks were observed centering on DNAJC10 (DnaJ heatshock protein family member C10)^[^ ^44^ ^]^ and RAB33B (member RAS oncogene family) (Figure S8a, Supporting Information).^[^ ^45^ ^]^ In miR‐487 target genes, MED28 (mediator complex subunit 28) was linked to the Mediator complex, a coactivator involved in the regulated transcription of nearly all RNA polymerase II‐dependent genes (Figure S9a, Supporting Information).^[^ ^46^ ^]^ In miR‐369‐5p, a network of potassium voltage‐gated channel family surrounding *KCNA1* was identified (Figure S10a, Supporting Information). For each of the miRNA*^INV^* target gene groups, there were genes that remain isolated. By combining all the target genes of the 3 miRNA*^INV^* s, we were able to expand the protein networks to link the nodes of DNAJC10, RAB33B, FLT1 (Fms Related Tyrosine Kinase 1)^[^ ^47^ ^]^ and EXOC5 (Exocyst Complex Component 5) (Figure S11a, Supporting Information).^[^ ^48^ ^]^ These sets of STRING identified novel protein networks critical to GBM invasion.

### 2.8 *KCNA1* is a Common Computational Target Gene of the miRNAs*^INV^*

**Figure 6:**
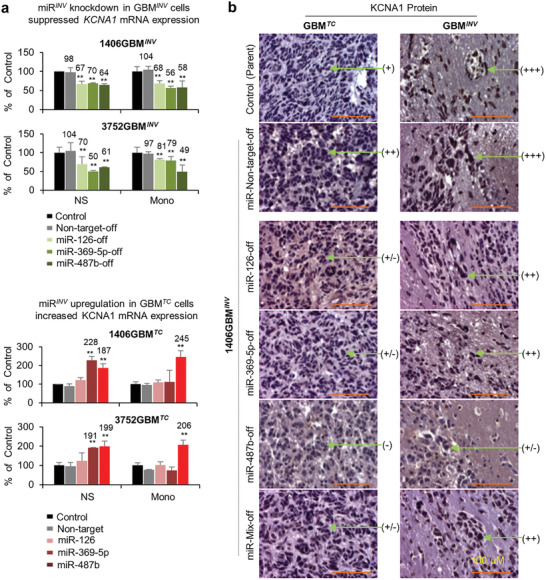
*KCNA1* is a molecular target of miRNA*^INV^*. a) Silencing miRNA*^INV^* led to decreased *KCNA1* mRNA expression in purified GBM*^INV^* cells from IC‐1406GBM (1406GBM ***^INV^***) and IC‐3752GBM (3752GBM ***^INV^***) grown as neurosphere (NS) and monolayer (Mono) (upper panel). Conversely, over‐expressing miRNA*^INV^* in GBM*^TC^* cells of these two models, resulted in elevated *KCNA1* mRNA expression in both culture conditions (lower panel). *KCNA1* expression was quantitated via qRT‐PCR (*P < 0.05). Data are shown as mean ± SD. b) KCNA1 protein expression was suppressed in vivo in the tumor core (GBM*^TC^*) and invasive front (GBM*^INV^*). GBM*^INV^* cells from IC‐1406GBM (1406GBM ***^INV^***) were transduced with Lentivirus‐miRNA off to silence miR‐126, ‐369‐5p, ‐487b alone (miR‐126‐off, miR‐369‐5p‐off, miR‐487‐off) and in combination (miR‐mix‐off), followed by implantation into the brains of SCID mice. KCNA1 protein expression was examined through IHC. Tumor cells receiving no (Control) or lentivirus‐non‐target‐off (miR‐Non‐target‐off) were included as references.

Since all 3 miRNA*^INV^* actively suppressed pGBM invasion, we examined if they shared common target gene(s) by comparing the target genes from each of the three miRNA*^INV^* ^s^ that were identified (detailed in the previous section). *KCNA1* was found to be the sole computational target gene shared by miR‐369‐5p, miR‐126, and miR‐487b (Figure 5a–c). *KCNA1* is potassium voltage‐gated channel subfamily A member 1, known to be involved in diverse physiological processes from repolarization of neuronal or cardiac action potentials to regulating calcium signaling and cell volume.^[^ ^49^, ^50^ ^]^ To functionally validate *KCNA1* as a molecular target of miRNA*^INV^*, we examined its expression after the loss‐of‐function and gain‐of‐function of the 3 miRNA*^INV^*. In GBM*^INV^* cells, which express high levels of *KCNA1* compared to GBM*^TC^* cells and normal brain tissue, silencing miR‐126, ‐369‐5p, and ‐487b significantly downregulated *KCNA1* (25–45% compared with non‐target control) in both neurosphere and monolayer cultures of IC‐1406GBM*^INV^* and IC‐3752GBM*^INV^* cells (**Figure** **6**a, top panel). Conversely, enhancing miRNA*^INV^* expression in GBM*^TC^* cells (in which *KCNA1* expression was originally low) of IC‐1406GBM*^TC^* and IC‐3752GBM*^TC^* increased *KCNA1* mRNA expression in neurospheres by miR‐369‐5p and ‐487b; in monolayer cells by miR‐487b and mild elevation by miR‐126 (Figure 6a, lower panel).

To validate *KCNA1* as a molecular target of miRNAs in vivo, *KCNA1* expression in the invasive pGBM cells were examined in IC‐1406GBM and IC‐3752GBM by IHC staining. Compared with strong (+++) *KCNA1* positivity in GBM*^INV^* cells that migrated into the normal brains, silencing miRNA*^INV^* (miR‐126, ‐369‐5p, and ‐487b) significantly reduced *KCNA1* expression to low (+/− to ++) levels (Figure 6b) in the invasive front, although the depth of invasion was significantly reduced after the silencing of miRNA*^INV^*.

Since a gene can be regulated by multiple miRNAs, we hypothesized that *KCNA1* would be further regulated (fine‐tuned) by additional miRNAs if it is critical for pGBM invasion. A reverse scan of the miRNAs differentially expressed by GBM*^INV^* and GBM*^TC^* cells in the current study revealed that *KCNA1* is the target (or putative target) gene redundantly regulated by 10/23 (47.8%) miRNA*^INV^* and 18/22 (81.8%) miRNA*^TC^* (Figure 5c), further supports a role of *KCNA1* in pGBM invasion.

### 2.9 *KCNA1* is Overexpressed in GBM*^INV^* Cells In Vivo in Patient‐Derived Orthotopic Xenograft Models and in Patient Glioblastoma Tumors

Although highly desired, examination of KCNA1 expression in the invasive front in human GBM patients is challenged by the difficulties of obtained normal brain tissues from patients. To further confirm KCNA1 over‐expression in the GBM*^INV^* cells, we compared its mRNA and protein levels between GBM*^INV^* and GBM*^TC^* cells in 6 PDOX models. Elevated (>1.5‐fold) *KCNA1* mRNA expression in GBM*^INV^* cells was detected in 4/6 models (Figure 5d). KCNA1 protein expression was subsequently analyzed in whole mouse brain sections through IHC. Compared with low (− to ++) levels of KCNA1 in GBM*^TC^* cells, strong (++ to +++) KCNA1 positivity (*KCNA1* ^+++^) was detected in GBM*^INV^* cells in 5/6 models (Figure 5e,f). Altogether, over‐expression of *KCNA1* mRNA and protein were detected in 4/6 models, while elevated expression of mRNA only was found in IC‐1621GBM and protein only in IC‐3752GBM*^TC^* (Figure 5e,f and Figure S12, Supporting Information). These data indicated that KCNA1 over‐expression, particularly at protein level, is frequent in the GBM*^INV^* cells.

To validate our discoveries in patient tumors, we extracted the RNAseq data from the IVY Atlas, where anatomic structures RNAseq was completed (in 120 samples from 10 tumors) in the leading edge as well as infiltrating tumor, cellular tumor, microvascular proliferation, and pseudopalisading cells around necrosis identified by H&E staining (https://glioblastoma.alleninstitute.org/static/home). Expression levels of the 239 unique target genes of miRNA*^INV^* were compared with the genes in the leading edges of patient GBMs (Table S5, Supporting Information). Among the 112 significantly (*P* < 0.05) over‐expressed miRNA*^INV^* target genes, 41 (36.6%) genes were discovered to be significantly elevated (*P* < 0.05) in the leading edges of patient GBM tumors found in the IVY project, including the KCNA1, which was 1.54‐fold (GBM*^INV^*/GBM*^TC^*) in the PDOX and 4.79‐fold in the leading edges of patient tumors (group 1 in Table S5, Supporting Information). Of the 127 significantly downregulated miRNA*^INV^* target genes, 42 genes (33%) were also significantly different between the leading edges and tumor mass (group 2, Table S5, Supporting Information). Some discrepancies between the current study and the patient tumor findings were also noted, included 13 (11.6%) genes that were over‐expressed in the PDOX invasive cells but significantly downregulated in the patient tumors, and 30 (23.2%) downregulated in PDOX invasive cells but upregulated in the patient GBM leading edges (group 3 and 4, Table S5, Supporting Information). Despite the differences of invasive GBM cell isolation approaches, it is very encouraging to see the validation of > 60% of the miRNA*^INV^* target genes in patient GBM leading edges.

### 2.10 Pharmacological Targeting of KCNA1 Suppresses Pediatric Glioblastoma Invasion In Vitro and In Vivo

**Figure 7:**
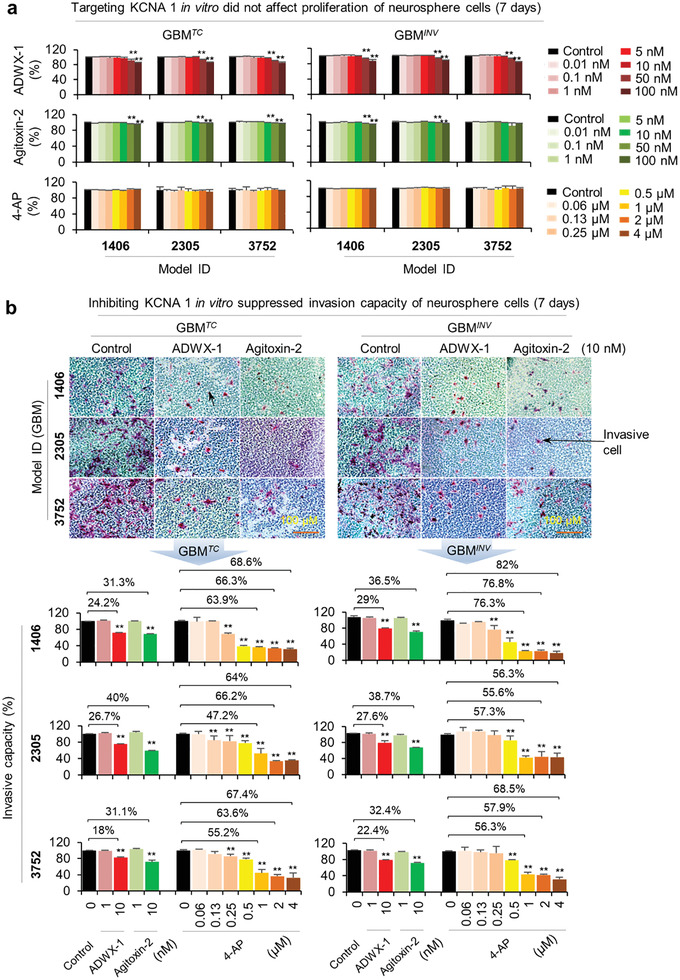
Pharmacological inhibition of *KCNA1* suppresses pGBM invasion in vitro. a) Impact of 3 *KCNA1* inhibitors on cell proliferation were examined in 3 pairs of neurosphere cultures of GBM*^TC^* and GBM*^INV^* cells derived from 3 PDOX models, IC‐1406GBM (1406), IC‐2305GBM (2305), and IC‐3752GBM (3752). Tumor cells were exposed to ADWX‐1 and Agitoxin‐2 at 0.01–100 nM for 1–14 days or 4‐AP (ranging from 0.06 to 4 µM) for 7, 10 days, respectively. Data on day 7 were shown (See left of Figure S13a–c, Supporting Information, for results of additional time points). Changes of cell proliferation were examined with CCK‐8 kit assay (*P < 0.05). b) *KCNA1* inhibitors suppressed in vitro invasion of both GBM*^TC^* and GBM*^INV^* cells derived from the 3 pGBM mouse models. Cells were treated with 1 and 10 nM of ADWX‐1 and Agitoxin‐2 for 3, 7, and 14 days or with 0.06, 0.13, 0.25, 0.5, 1, 2, and 4 µM of 4‐AP for 7, 10 days. *KCNA1* inhibitors also examined for changes of cell invasion using the CytoSelect 24‐Well Cell Invasion Assay (top panel). Data from the treated groups were normalized to the untreated (Control). Results from 7‐day treatment of 3 *KCNA1* inhibitors were presented (lower panel) (*P < 0.05), and those from other time points summarized in supplemental right of Figure S13a,b, Supporting Information. Data are shown as mean ± SD.

To explore the potential of KCNA1 inhibition in blocking GBM invasion, we treated 3 pairs of 3D neurosphere cultures of GBM*^TC^* and GBM*^INV^* cells (shown to have the strongest invasion capacity in vitro) with 3 KCNA1 inhibitors. Our aim was to confirm that the effects were not restricted to a single tumor model and could be reproduced by multiple inhibitors (i.e., not caused by off‐target activities). 3 KCNA1 inhibitors were employed, including ADWX‐1 and Agitoxin‐2 at 0.01–100 nM and 4‐aminopyridine (4‐AP) at 0.06–4 µM. For ADWX‐1 and Agitoxin‐2, only high doses (50 and 100 nM) caused minor suppression of cell proliferation (<20%) after 14 days’ exposure (**Figure** **7**a and Figure S13a,b, Supporting Information). 4‐AP did not affect cell proliferation even at the highest dose (4 µM) after 7‐ and 10‐days’ exposure (Figure 7a and left of Figure S13c, Supporting Information).

The effects on cell invasion were thus investigated with doses that did not affect cell proliferation by focused on the neurosphere cultures that have exhibited a stronger invasive capacity than the monolayer cells in vitro. While no significant changes were noted in the 1 nM groups, treatment with ADWX‐1 and Agitoxin‐2 at 10 nM resulted in significant inhibition of cell invasion in GBM*^TC^* and GBM*^INV^* cells starting from day 3 through 14 (Figure 7b and right of Figure S13a,b, Supporting Information). Agitoxin‐2 was more potent than ADWX‐1, exhibiting stronger inhibition in GBM*^INV^* cells 1.5 ± 0.3‐fold higher than that in GBM*^TC^* cells (35% ±1.7%, vs 24.1% ± 6.7%). 7 days of treatment led to peak inhibition in both GBM*^INV^* (35.9% ± 4.5%) and GBM*^TC^* (34.1% ± 4%) cells, while prolonged exposure to14 days did not further inhibit cell invasion. Since 4‐AP did not affect cell proliferation, we examined its activities in a more detailed time‐ (7–10 days) and dose‐setting (0.06, 0.13, 0.25, 0.5, 1, 2, and 4 µM). Significant dose‐dependent inhibition of cell invasion up to 82% at 4 µM, the highest among 3 inhibitors, was observed starting from day 7 and lasted to day 10 (Figure 7b and Figure S13c, Supporting Information). The overall levels of suppression in GBM*^INV^* neurosphere cells were higher than that in the matching GBM*^TC^* cells. In summary, invasion was suppressed by all 3 KCNA1 inhibitors in 3 pairs (*n* = 6) of pGBM neurosphere cultures.

**Figure 8:**
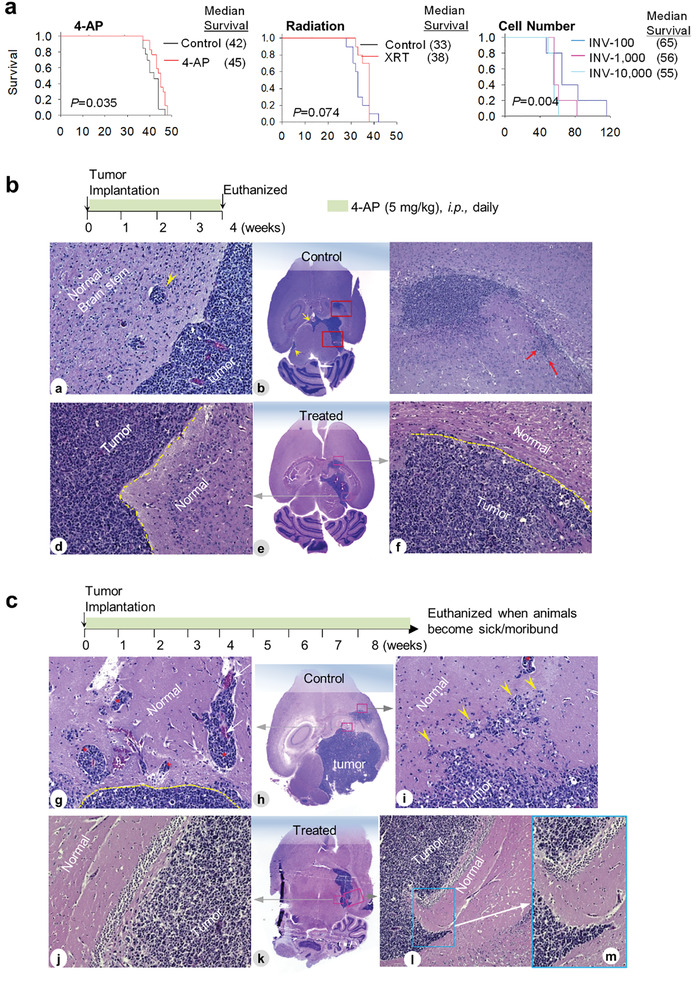
Suppression of pGBM invasion by 4‐AP in vivo in IC‐3752GBM. a) Log‐rank analysis of animal survival times. Mice (*n* = 10–15/group) bearing IC‐3752GBM were treated with 4‐AP daily (left panel) or fractionated radiation (2 Gy/day × 5 days) or injected with different cell numbers and compared with those administered with vehicle (PBS). b) Changes of pGBM invasion during 4‐AP treatment. Mouse brains were harvested on 4 weeks post drug treatment (top panel) followed by H&E staining of paraffin sections. In the untreated tumors (a–c), para‐vascular invasion (a, arrow), CSF spread (b, arrow), and deep invasion (c, arrow) were prominent. Tumors treated with 4‐AP treatment (d–f) for four weeks exhibited sharp margins between tumor and normal tissues (e and f, dotted yellow lines). c) Reduced invasion after long‐term in vivo treatment with *KCNA1* inhibitor 4‐AP. The animals were continuously treated daily until they were euthanized due to sickness or moribund (top panel). Representative images from the untreated control (g–i) showing large foci of para‐vascular migration (g, *) from tumor into normal brains, and diffuse single cell invasion (i, arrowhead) as compared with well‐defined tumor and normal border with very limited or no tumor invasion (j–m). Magnification: 10× (c and i), 20× (all others).

Compared with high molecular weight of ADWX‐1 (*M*_W_ = 4071.86) and Agitoxin‐2 (*M*_W_ = 4090.87), which are >ninefold higher than the theoretical molecular weight threshold of 450 Dalton for molecules able to pass through the BBB,^[^ ^51^ ^]^ 4‐AP is very small (*M*_W_ = 94.11) and shown to be able to penetrate the BBB.^[^ ^52^, ^53^ ^]^ Additionally, 4‐AP is a FDA approved drug for Lambert‐Eaton myasthenic syndrome (a rare autoimmune disorder characterized by muscle weakness of the limbs) and multiple sclerosis.^[^ ^54^ ^]^ We therefore prioritized 4‐AP to assess its anti‐invasion and therapeutic efficacy. Mice bearing IC‐3752 GBM*^INV^* ^3^ were treated with 4‐AP (5 mg kg^−1^) daily after tumor implantation. Although 4‐AP is potent convulsant and can generate seizures in animals,^[^ ^55^ ^]^ we only observed signs of excitement (increased activities) without significant (>15%) loss of body weight. Median animal survival times in IC‐3752GBM models were prolonged from 42 days in the vehicle group to 45 days in the treatment group (*P* = 0.035; **Figure** **8**a). This extension was equivalent to the efficacy of fractionated radiation (2 Gy/day × 5 days). Since reducing implanted tumor cells from 10 000 to 1000 per mouse did not change animal survival times, which highlighted the highly malignant and progressive nature of IC‐3752GBM, the significantly extended animal survival times indicated the efficacy of 4‐AP as a single agent.

To examine if the reduction of pGBM invasion mediated the extended animal survival times (since 4‐AP did not affect pGBM proliferation in vitro), we euthanized 2 mice at the end of 4‐week drug administration (Figure 8b). Compared with the frequent invasion (single cell, perivascular) and CSF spread in the untreated mice (Figure 8a–c), the treated tumors exhibited a sharp margin between the normal tissue and the tumor with low to no local invasion (Figure 8b,d–f). Similar reduction of tumor invasion was confirmed in the remnant tumors of mice in the survival group in which the treated GBM tumors were clearly circumscribed with few invasive cells and dramatically reduced CSF spread (Figure 8c), although the tumor sizes were similar between the treated and the control groups. These data demonstrated the in vivo inhibition of pGBM invasion by a single agent 4‐AP, which in turn has contributed to the significantly extended animal survival times.

## 3 Discussion

In this study, we utilized a novel set of highly invasive PDOX pGBM models to harvest and functionally validate matching pairs of GBM*^INV^* and GBM*^TC^* cells. Through global miRNA profiling, we identified a novel set of miRNAs that were differentially expressed between GBM*^INV^* (miRNA*^INV^*) and GBM*^TC^* (miRNA*^TC^*) cells of different molecular subtypes. Silencing the top 3 miRNA*^INV^* (miR‐126, ‐369‐5p, and ‐487b) led to significant suppression of GBM*^INV^* cell invasion (without affecting cell proliferation) in vitro and blocked pGBM invasion in vivo in the brains of SCID mice, thereby establishing these miRNA*^INV^* as novel drivers of pGBM invasion. Mechanistically, we confirmed *KCNA1* as the sole common computational target gene of the 3 miRNAs*^INV^*. Using a set of KCNA1 inhibitors, we demonstrated that pharmacological targeting of KCNA1 significantly suppressed GBM*^INV^* cell invasion in vitro, blocked GBM invasion in vivo and prolonged animal survival times in a highly invasive pGBM model.

Pediatric GBM invasion is a complex biological process involving a dynamic interplay between tumor cells and surrounding normal brain tissues. Due to the risk of severe neurological deficits, extended surgical resection of normal brain tissues is rarely attempted, making it nearly impossible to obtain GBM*^INV^* cells for biological studies of GBM invasion. Our PDOX models that replicated the diffuse invasion of GBM have thus provided an opportunity to overcome this barrier by allowing for the isolation of purified matching pairs of GBM*^INV^* and GBM*^TC^* cells. Our finding of elevated invasive capacity of GBM*^INV^* cells in vitro and in vivo showed that the GBM invasion is not a random event but a capacity endowed with a selected subpopulations of tumor cells. This discovery demonstrated the power and highlighted the importance of using functionally‐ and biologically‐ accurate source cells (i.e., GBM*^INV^*) for future biological studies and pre‐clinical drug testing for targeting pGBM invasion.

Identifying molecular drivers of pGBM invasion is an important step in developing new anti‐invasion therapies. Given the critical roles of miRNA in regulating critical and complex biological activities, we performed global miRNA profiling of matching pairs of GBM*^INV^* and GBM*^TC^* cells and identified a novel miRNA signature overexpressed in the invasive front (miRNA*^INV^*) and in the tumor core (miRNA*^TC^*), respectively. In addition to miRNAs that have been previously associated with tumor invasion/metastasis, such as miR‐126,^[^ ^40^, ^41^ ^]^ we also discovered novel miRNAs that may contribute to GBM invasion. Of note, many of these differentially expressed miRNA*^INV^* and miRNA*^TC^* would have been missed if only normal brain tissues were utilized as references, which again underscores the importance of using functionally‐accurate cell sub‐populations to address critical biological questions.

To validate the role of miRNA*^INV^* candidates in driving pGBM invasion, we selected 3 miRNA*^INV^* for in vitro and in vivo functional studies, including 2 newly identified miRNA*^INV^* (miR‐369‐5p and ‐487b) and miR‐126, whose role in invasion/metastasis of multiple human cancers has been previously reported.^[^ ^40^, ^41^ ^]^ Silencing these miRNAs led to significant inhibition of GBM*^INV^* cell invasion without affecting cell proliferation in vitro, particularly in the 3D neurospheres, in 2 distinct pGBM cell cultures, indicating the functional specificity of these miRNA*^INV^* in pGBM invasion. Subsequent in vivo confirmation of blocked invasion in mouse brains after miRNA silencing provided strong functional evidence to support the role of these miRNA*^INV^* in driving pGBM invasion.

Since silencing all 3 candidate miRNA*^INV^* did not completely eliminate pGBM invasion, additional drivers may exist. Indeed, we have discovered a total of 23 miRNA*^INV^* and 20 miRNA*^TC^* that may regulate pGBM invasion, which highlights the need for additional functional studies. However, even converting a diffusely invasive pGBM into a tumor with “limited invasive capacity,” for example, infiltrating only several mm into surrounding normal brain parenchyma, may still improve the likelihood of complete surgical resection and overall survival in children with GBM.

Although the function of many miRNAs is still unknown, our integrated analysis of miRNA*^INV^* and global gene expression of the matching GBM*^INV^* and GBM*^TC^* cells linked differentially expressed miRNA with their target genes and pathways. While each of the 3 miRNA*^INV^* affected distinct canonical pathways, they shared some overlapping functions in disease and biology as well as molecular/cellular and physiological system development, thereby supporting the notion that they collectively promoted pGBM invasion. Our discovery of *KCNA1* as a commonly shared computational target gene by the 3 miRNA*^INV^* not only suggested a new biological node of miRNA*^INV^* regulation of pGBM invasion but also provided a therapeutic target. Validation of *KCNA1* over‐expression (together with an additional 40 upregulated miRNA*^INV^* target genes) in the leading edges of a precious set of carefully prepared patient GBM tumors provided much‐needed data to support the role KCNA1 in GBM invasion and our strategy in understanding GBM invasion. Although miRNAs usually downregulate target genes, there are reports showing that miRNAs can also positively regulate gene expression.^[^ ^56^, ^57^ ^]^ *KCNA1* is a potassium voltage‐gated channel subfamily A member 1 involved in diverse physiological processes from repolarization of neuronal or cardiac action potentials to regulating calcium signaling and cell volume.^[^ ^49^, ^50^ ^]^ It is a newly identified molecular marker for Group 4 medulloblastoma^[^ ^58^ ^]^ and is involved in breast cancer proliferation and regulation of oncogene‐induced senescence and transformation.^[^ ^59^ ^]^ Since channel proteins can be accessed from the extracellular milieu,^[^ ^60^ ^]^ *KCNA1* as a therapeutic target potentially allows for the use of lower drug doses thus decreased toxicities. Indeed, a series of *KCNA1* (also known as Kv) inhibitors have been developed.^[^ ^50^ ^]^ In addition to show anti‐invasion activities of 3 chemical inhibitors of KCNA1 in 3 sets of GBM*^INV^* cells, we demonstrated that 4‐AP, the smallest KCNA1 inhibitor that can penetrate the BBB,^[^ ^52^, ^53^ ^]^ effectively blocked pGBM invasion in vivo and significantly prolonged animal survival times in a highly invasive pGBM PDOX model. It is particularly encouraging that 4‐AP as single agent was able to extend animal survival times similar to that produced by fractionated radiation therapy. Although the overall level of survival time extension was not high (as 4‐AP did not suppress cell proliferation), inhibition of GBM invasion can improve the chances of completed tumor resection, thereby preventing tumor recurrence. Given the mild to moderate toxicity profiles, the recently identified neuroprotective properties, and the commercially available tablets for sustained release and long‐term administration in patients,^[^ ^52^, ^53^, ^54^, ^61^ ^]^ 4‐AP represents an ideal candidate drug for repurposing to target GBM invasions.

In summary, we demonstrated that not all pGBM cells have equal capacity of invasion (GBM*^TC^* cells are far less invasive than GBM*^INV^* cells), identified a novel miRNA*^INV^* signature through direct analysis of six matching pairs of biologically accurate GBM*^INV^* and GBM*^TC^* cells, completed functional validation of 3 miRNA*^INV^* (miR‐126, ‐369‐5p, and ‐487b) in driving pGBM invasion, discovered *KCNA1* as a druggable molecular target of the miRNA*^INV^*, and confirmed the pre‐clinical therapeutic efficacy of KCNA1 inhibitors as novel anti‐invasion agents both in vitro and in vivo. Our strategy of isolating matching pairs of biologically accurate invasive and tumor core cells is applicable to most of human cancer studies and can potentially cause a paradigm shift in the study of cancer invasion and metastasis.

## 4 Experimental Section

### Patient‐Derived Orthotopic Xenograft Mouse Models

Freshly resected brain tumor specimens were collected from six children undergoing surgery at Texas Children's Hospital in Houston, TX (Figure 1a). Signed informed consent was obtained from the patient or legal guardian prior to sample acquisition in accordance with our local Institutional Review Board (IRB) approved protocol. NOD/SCID mice were bred and housed in a specific pathogen‐free animal facility at Texas Children's Hospital. All animal experiments were conducted using an Institutional Animal Care and Use Committee (IACUC)—approved protocol as described previously.^[^ ^14^, ^15^, ^62^ ^]^ Tumor tissues were mechanically dissociated within 60 min of surgical removal. After the cell suspensions were passed through 40 and 100 µm cell strainers, viable tumor cells were dissociated into single cells, and small clumps (≈5–10 cells) were counted with Trypan blue staining. Tumor cells (1 × 10^5^) were then suspended in 2 µL of culture medium and injected into mice brains 1 mm to the right of the midline, 1.5 mm anterior (for intra‐cerebral tumors) to the lambdoid suture, and 3 mm deep via a 10 µL 26‐gauge Hamilton Gastight 1701 syringe needle (Figure 1b). The animals were monitored daily until they developed signs of neurological deficits or became moribund, at which time they were euthanized.

### Immunohistochemistry

IHC was performed using a Vectastain Elite kit (Vector Laboratories, Burlingame, CA) as described previously.^[^ ^15^, ^30^ ^]^ Primary antibodies included the human‐specific mitochondria monoclonal antibody (1:50) (MAB1273MI, fisher scientific) and mouse anti‐glial fibrillary acidic protein (GFAP) (1:200) (M0761, AGILENT TECHNOLOGIES INC), VIM (1:200) (M0725, Dako North America,), MAP‐2 (1:200) (AB7756, Abcam Inc), Ki67 (1:20) (ab833‐500, Abcam Inc), and rabbit anti‐Nestin (NES) (1:500) (ABD69, EMD Milipore). After slides were incubated with primary antibodies for 90 min at room temperature, the appropriate biotinylated secondary antibodies (1:200) were applied and incubated for 30 min. The final signal was developed using the 3,3’‐diaminobenzidine substrate kit for peroxidase. IHC staining was assessed by combining the intensity and extent of immunopositivity.^[^ ^15^, ^30^ ^]^

### In Vivo Quantitative Analysis of Pediatric Glioblastoma Invasion

Quantitative analysis of pGBM invasion was performed using whole mouse brains that were harvested when the tumor‐bearing mice became moribund and then fixed and paraffin embedded. Serial paraffin sections were then subjected to standard H&E and IHC staining with human‐specific antibodies against MT and VIM to positively identify human pGBM cells in the mouse brain and antibodies recognizing both human and mouse vWF to identify micro‐blood vessels. The modes of GBM invasion, that is, single cell migration, along neural fibers, infiltrating to CSF and following micro‐blood vessels, were identified under the microscope (10–40×). To achieve a comprehensive and quantitative evaluation of GBM invasion, the digitally captured gross and microscopic images (4–40×) on the largest cross sections (mean ± SD) from at least 3 (3.5 ± 1.2) whole mouse brains were analyzed used a straight line reticle (eyepiece micrometer) to measure the distances (in µM) of migration.^[^ ^29^ ^]^ Representative images were also taken and analyzed with ImageJ software to quantify the longest distance between the leading edge of invasive tumor cells and the edge of the primary tumor mass.

### Isolation of Functionally Validated Invasive (GBM*^INV^*) and Tumor Core (GBM*^TC^*) Cells

To isolate matched pairs of GBM*^INV^* cells and non‐invading GBM*^TC^* cells, freshly harvested whole mouse brains were placed on a mouse brain matrix and sliced at 1 mm thickness into 10–12 slices (Figure 2a). These brain slices were then submerged in cold (4 °C) growth medium and the tumor core/mass (GBM*^TC^* cells) was subsequently dissected from the “normal” mouse brain tissues (containing GBM*^INV^* cells) with a surgical scalpel under a surgical microscope (Figure 2b).^[^ ^32^, ^33^, ^34^ ^]^ The 2 compartments of tumor tissues, the “normal” brain (containing GBM*^INV^* cells) and the tumor mass (with GBM*^TC^* cells) from the same mouse brain were pooled and mechanically dissociated into single cell suspensions using Gentle Dissociator (Miltenyi). After passing through a stack of cell strainers (100 and 40 µm), viable tumor cells were counted after being stained with trypan blue.

### Florescence Activated Cell Sorting

To isolate pure human GBM*^INV^* and GBM*^TC^* tumor cells without mouse cell contamination, particularly from the “normal” mouse brain fractions, cell suspensions were incubated with FITC‐conjugated monoclonal antibodies against human HLA‐ABC (BD Biosciences, 555552, Franklin Lakes, NJ), and an APC‐conjugated cocktail of monoclonal antibodies against mouse major histocompatibility antigen for 15 min. The mouse antibody cocktail included APC‐conjugated monoclonal antibodies against mouse CD24, CD90, CD117, CD133 (BD Biosciences, Franklin Lakes, NJ), or mouse major histocompatibility antigen (MHC) H2 haplotype (H‐2 Db‐APC). The stained cells were then washed and subjected to FACS (Figure 2c and Figure S2, Supporting Information) to gate out mouse cells and dead cells (propidium iodine high).

### Whole Genome miRNA Profiling

Whole genome miRNA profiling was completed using a two‐card set of TaqMan Array MicroRNA Cards (Cards A and B) for a total of 754 unique assays specific to human miRNAs (Invitrogen) following the manufacturer's instructions. The presence of the target was detected in real time through cleavage of the TaqMan probe by polymerase 5′–3′ exonuclease activity. Briefly, total RNAs were extracted using the *mir*Vana miRNA isolation kit. To synthesize single‐stranded cDNA, 500 ng of total RNA were reverse transcribed using the Taqman MicroRNA reverse transcription kit and the Megaplex RT Primers on a 7900HT real‐time PCR system. 6 µL of Megaplex RT product was then mixed with 450 µL of TaqMan Universal PCR Master Mix and 444 µL of nuclease‐free water and before being dispensed in 100 µL aliquots into each port of the array card. The card was briefly centrifuged, sealed, and loaded on to the Applied Biosystems 7900HT fast real‐time PCR system for amplification. Relative quantitation of target miRNA expression levels was performed through comparative C_T_ analysis using the same threshold setting for all arrays.

### Global Gene Expression Profiling

Global gene expression profiling was performed using Affymetrix U133 Plus 2.0 chips (Affymetrix, Santa Clara, CA). For RNA purification and cDNA amplification, total RNA was extracted with RNeasy (Qiagen), processed, and cDNA amplified using the Affymetrix RNA amplification and biotinylation kit. Fragmented and biotinylated cDNA was then hybridized to Affymetrix U133 Plus 2.0 chips following the manufacturer's instructions. The stained chips were then scanned using the Affymetrix GeneChip scanner 3000. The data file was imported to Bioconductor and Partek for data analyses and deposited at the Gene Expression Omnibus (GEO) Web site.

Microarray data analysis and quality control of the Affymetrix U133 Plus 2.0 gene‐expression chips were performed using the BioConductor package affyQCReport. *β*‐Actin and GAPDH ratios, as well as, signal distribution, were assessed to determine the outlier cases. Normalization and probe set summarization was done in BRB‐Arraytools (http://linus.nci.nih.gov/BRB‐ArrayTools. html) using the Robust Multichip Average algorithm. Hierarchical clustering was performed using centered correlation and average linkage. The significance analysis of microarrays algorithm was used for analysis of differential expression, with a false discovery rate of 0.01. Heatmaps and other graphics were created using Multi‐Experiment Viewer, part of the TM4 Microarray Software Suite. The log intensity values were exported and analyzed in Bioconductor.^[^ ^63^ ^]^ To perform clustering, all samples were included using the hierarchical clustering algorithm provided in the “stats” R library. To determine the correlations, all elements were used to calculate the correlation coefficient (*R* ^2^ values) of all groups of samples. Pair‐wise comparison was done between pGBM cells from the invasive front, invasive rim, and the central core to identify the deregulated genes.

### DNA Methylation Profiling

DNA methylation profiling of the six PDOX models was performed using Illumina Infinium Human Methylation450 arrays as previously described.^[^ ^27^ ^]^ Tumors were classified based on their DNA methylation profiles using the DKFZ brain tumor methylation classifier (http://www.molec ularneuropathology.org), which is based on a reference cohort of 2801 CNS tumors and normal tissues representing 82 distinct tumor and 9 distinct non‐neoplastic brain methylation classes.^[^ ^27^ ^]^

### Infection of Pediatric Glioblastoma Cells with Lentiviral miRNAs

To silence the expression of miRNA, five miRNA‐specific lentiviral transduction particles were purchased from Applied Biological Materials Inc. (ABM, MC, Canada), including LentimiRa‐Off‐has‐miR‐126 (hereafter referred as Lenti‐miR‐126‐off), LentimiRa‐Off‐has‐miR‐487b (Lenti‐miR‐487b‐off), LentimiRa‐Off‐has‐miR‐369‐5p (Lenti‐miR‐369‐5p‐off), LentimiRa‐Off‐has‐miR‐589 (Lenti‐miR‐589‐off), and LentimiRa‐Off‐has‐miR‐185 (Lenti‐miR‐185‐off). To increase miRNA expression, an additional five lentiviruses were obtained, including LentimiRa‐GFP‐has‐miR‐126 (Lenti‐miR‐126), LentimiRa‐GFP‐has‐miR‐487b (Lenti‐miR‐487b), Lenti‐miRa‐GFP‐has‐miR‐369‐5p (Lenti‐miR‐369‐5p), LentimiRa‐GFP‐has‐miR‐589 (Lenti‐miR‐589), and LentimiRa‐GFP‐has‐miR‐185 (Lenti‐miR‐185). Lentiviruses expressing GFP (Lenti‐off‐GFP and Lenti‐GFP) were used to monitor transduction efficiency, and those containing no miRNA or non‐target miRNA of Lenti‐III‐miR‐Off Control (Lenti‐off‐non‐target‐GFP) and Lenti‐III‐miR‐GFP Control (Lenti‐nontarget‐GFP) were included as non‐target controls. To silence or activate target miRNAs, purified GBM*^INV^* and GBM*^TC^* cells were seeded in traditional FBS‐containing DMEM and serum‐free media supplemented with EGF (50 ng mL^−1^), bFGF (50 ng mL^−1^), B2 (1×), and N27 (1×). The culture media was removed 24 h later, replaced with 100 µL of Polybrene (2 µg mL^−1^) media mixture, and exposed to lentiviral‐miRNAs for 72 h (MOI 1:1). The infection efficiency was monitored by flow cytometry, florescent microscopy examination of GFP expression, and quantitative RT‐PCR (RT‐qPCR). GFP^−^positive cells were selected by puromycin (1 µg mL^−1^) for 4 days before being counted and seeded to 96‐well plates.

### Quantitative RT‐PCR

Total RNAs enriched with miRNAs were extracted using miRNeasy Mini Kit (Qiagen) following the manufacturer's instructions and reverse transcribed into cDNA using miRNA cDNA Synthesis Kit (G270, Applied Biological Materials Inc, Richmond, BC, Canada). RT‐qPCR was performed with a miScript SYBR PCR kit (Qiagen) using specific primers for miR‐126, miR‐369‐5p, miR‐487b, miR‐185, and miR‐589 (Applied Biological Materials, Inc., Richmond, BC, Canada). The relative levels of miRNA transcripts were normalized to SNORD44, a control miRNA recommended by the manufacturer (Applied Biological Materials, Inc.); *KCNA1* mRNA expression was quantitated with qRT‐PCR (forward primer 5’ CTGAGCAGGAAGGAAACCAG3’ and reverse primer 5’ CCTTAGAGTGGCGGGAGAG3’)^[^ ^16^ ^]^ and normalized to *GAPDH* (forward primer 5’AAGGTGAAGGTCGGAGTCAA3’ and reverse primer 5’ AATGAAGGGGTCATTGATGG3’) through standard ΔΔCt method as described previously.^[^ ^14^ ^]^

### In Vitro Cell Invasion Assay

A CytoSelect 96‐well cell invasion assay (Cell Biolabs, San Diego, CA) was used following the manufacturer's instructions. Briefly, 1 × 10^5^ cells in starvation medium were plated onto the upper chamber of an 8 µm pore polycarbonate membrane and exposed to a lower chamber filled with FBS growth or/and serum‐free media. After incubation at 37 °C for 24 h, the inserts were taken out, and the cells that migrated through the membrane were stained with CyQuant GR dye solution for 20 min (CBA‐112, Cell Biolabs) for quantification. The intensity of fluorescence was measured using a Synergy 2 Microplate Reader (excitation at 485 nm and emission at 528 nm) (Synergy, BioTek).

### In Vitro Treatment with ADWX‐1, Agitoxin‐2, and 4‐AP

To determine the time‐ and dose‐dependent effects of 3 *KCNA1* inhibitors of ADWX‐1, Agitoxin‐2, and 4‐AP, paired GBM*^TC^* and GBM*^INV^* cells from IC‐1406GBM (1406), IC‐2305GBM (2305), and IC‐3752GBM (3752) were seeded into 96‐well plates in quadruplicates and exposed to 7 doses of ADWX‐1 and Agitoxin‐2 (ranging from 0.01 to 100 nM), of 4‐AP (ranging from 0.06 to 4 µM) or vehicle control, respectively. Cell viability was measured at day 1, 3, 7, 10, and 14 for ADWX‐1 and Agitoxin‐2 and at day 7 and 10 for 4‐AP using the cell counting kit‐8 (CCK8; Dojindo Molecular Technologies) as ref. ^[^ ^15^, ^30^ ^]^ and cell invasion capacity was detected by invasion assay at the meantime as described in method above.

### In Vivo Treatment of Patient‐Derived Orthotopic Xenograft Tumors with 4‐AP

4‐AP was dissolved in PBS to achieve a final concentration of 0.5 mg mL^−1^. The same day of intracerebral IC‐3752 GBM*^INV^* tumor cell implantation, mice were intraperitoneal administrated with 4‐AP (5 mg kg^−1^) once daily till the endpoint of mice. To determine any survival benefits from 4‐AP treatment, the mice were monitored daily until they developed signs of neurologic deficit or became moribund, at which time they were euthanized, and their brains were removed for analysis.

### Analysis of the Protein Interaction Network

STRING (http://www.string‐db.org), a database and a tool for predicting direct and indirect protein–protein interactions was utilized. This database is derived from the following sources: previous knowledge, high‐throughput experiments, genomic context, and conserved co‐expression. In addition to the miRNA target genes, 20 necessary proteins were also inputted into STRING to generate an output network of the protein‒protein interactions between the targeted genes and those necessary added proteins.

### Statistical Analysis

Values were presented as mean and standard deviation (mean ± SD) with potential differences analyzed with the Student's *t*‐test and two‐way ANOVA. *P* < 0.05 is considered significant. Changes of animal survival times were examined through log‐rank analysis using Sigmaplot 14.

## Conflict of Interest

The authors declare no conflict of interest.

## Author Contributions

Y.H. and L.Q. contributed equally to this work. X.‐N.L. and Y.H. conceived the project; X.‐N.L. led the experimental design; Y.H., L.Q., M.K., Y.D., S.X., H.Z., H.L., S.Z., P.B., and X.‐N.L. performed the in vivo studies; S.B., M.K., and S.M.P. led the molecular sub‐ classification of PDOX models; Y.H., L.Q., F.K.B., and X.‐N.L. carried out the miRNA profiling and in vitro studies; Y.H., L.F.H., L.Q., and X.‐N.L. performed the computational studies; J.M.S., C.K.M., J.Y., M.C., D.W.P., Z.W., and Y.Z. participated in the experiment design and data analysis; A.A. provided the histopathology evaluation of the in vivo models; L.P., Z.W., Y.Z., and W.‐Y.T. provided the technology guidance of in vivo study; S.B., M.K., and S.M.P. contributed the analysis of gene profiling; X.‐N.L. and L.Q. wrote the manuscript; and all authors reviewed the manuscript.

## Supporting information

Supporting Information

Supplemental Table 1‐5

## Data Availability

The data that support the findings of this study are available from the corresponding author upon reasonable request.
