# Supplementary material for: Spatial Dissection of Invasive Front from Tumor Mass Enables Discovery of Novel microRNA Drivers of Glioblastoma Invasion
Source: Adv Sci (Weinh). 2021 Nov 1;8(23):2101923. doi: 10.1002/advs.202101923 (PMC8655179; doi:10.1002/advs.202101923)
Supplement: Supplementary file 1 — Supporting Information [file ADVS-8-2101923-s002.pdf]

© 2021 Wiley-VCH GmbH

ADVANCED SCIENCE
Open Access

Supporting Information

for *Adv. Sci.*, DOI: 10.1002/advs.202101923

Spatial Dissection of Invasive Front from Tumor Mass Enables Discovery of Novel microRNA Drivers of Glioblastoma Invasion

Yulun Huang[1,2‡], Lin Qi[2,3‡], Mari Kogiso[2], Yuchen Du[2,3], Frank K. Braun[2], Huiyuan Zhang[2], Lei F. Huang[4], Sophie Xiao[3], Wan-Yee Teo[5], Holly Lindsay[2], Sibo Zhao[2], Patricia Baxter[2], Jack MF Su[2], Adekunle Adesina[6], Jianhua Yang[2], Sebastian Brabetz[7,8], Marcel Kool[7,8], Stefan M. Pfister[7,8,9], Murali Chintagumpala[2], Laszlo Perlaky[2], Zhong Wang[1], Youxin Zhou[1], Tsz-Kwong Man[2], Xiao-Nan Li[2,3]*

Supplemental Figures

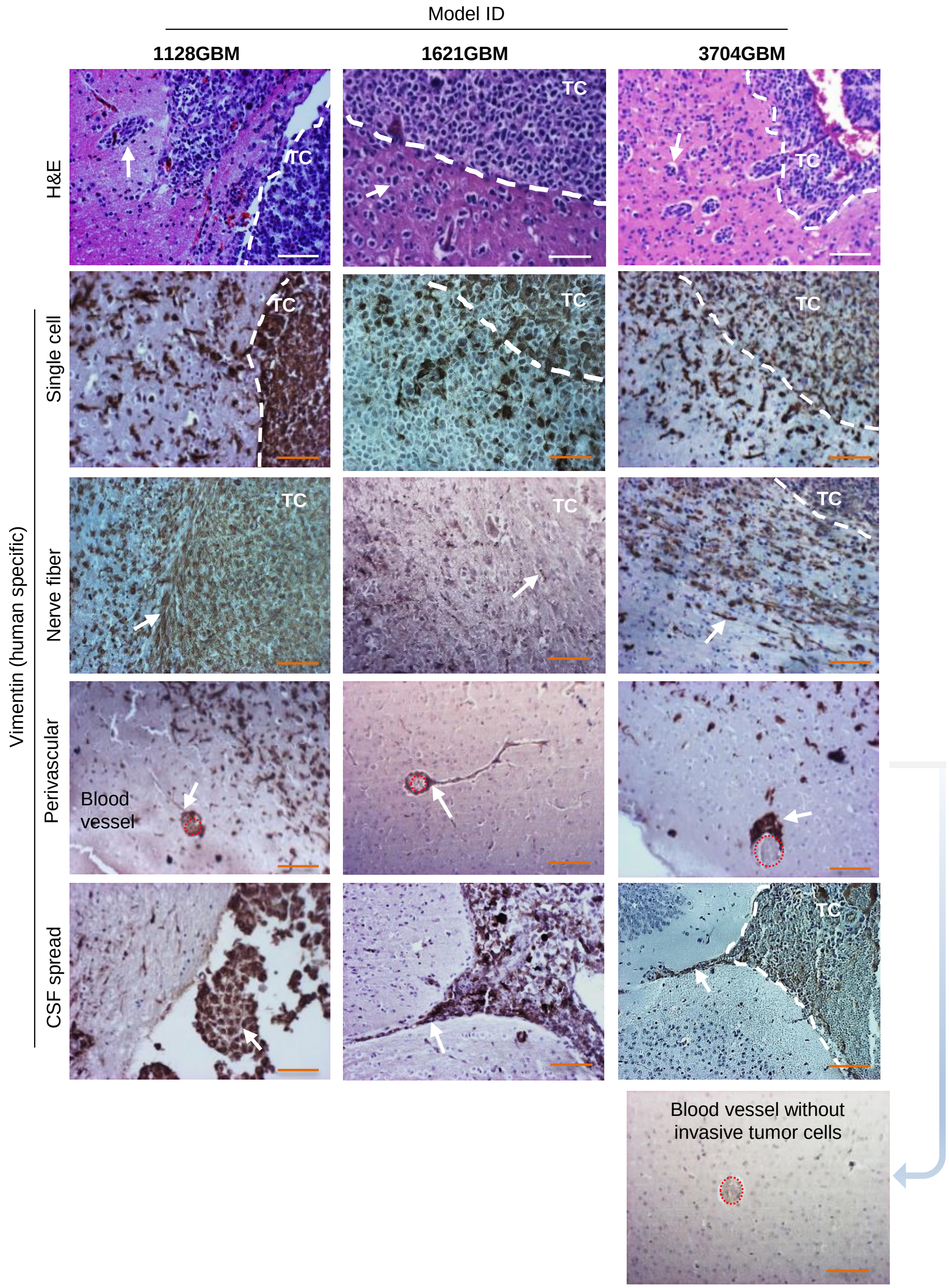

Suppl. Figure 1. Replication of diffuse invasion of pGBMs in PDOX models. H&E and Immunohistochemical staining vimentin showing different pattern and route of invasive growth of the remaining three pGBM PDOX models, IC-1128GBM, IC-1621GBM and IC-3704GBM.

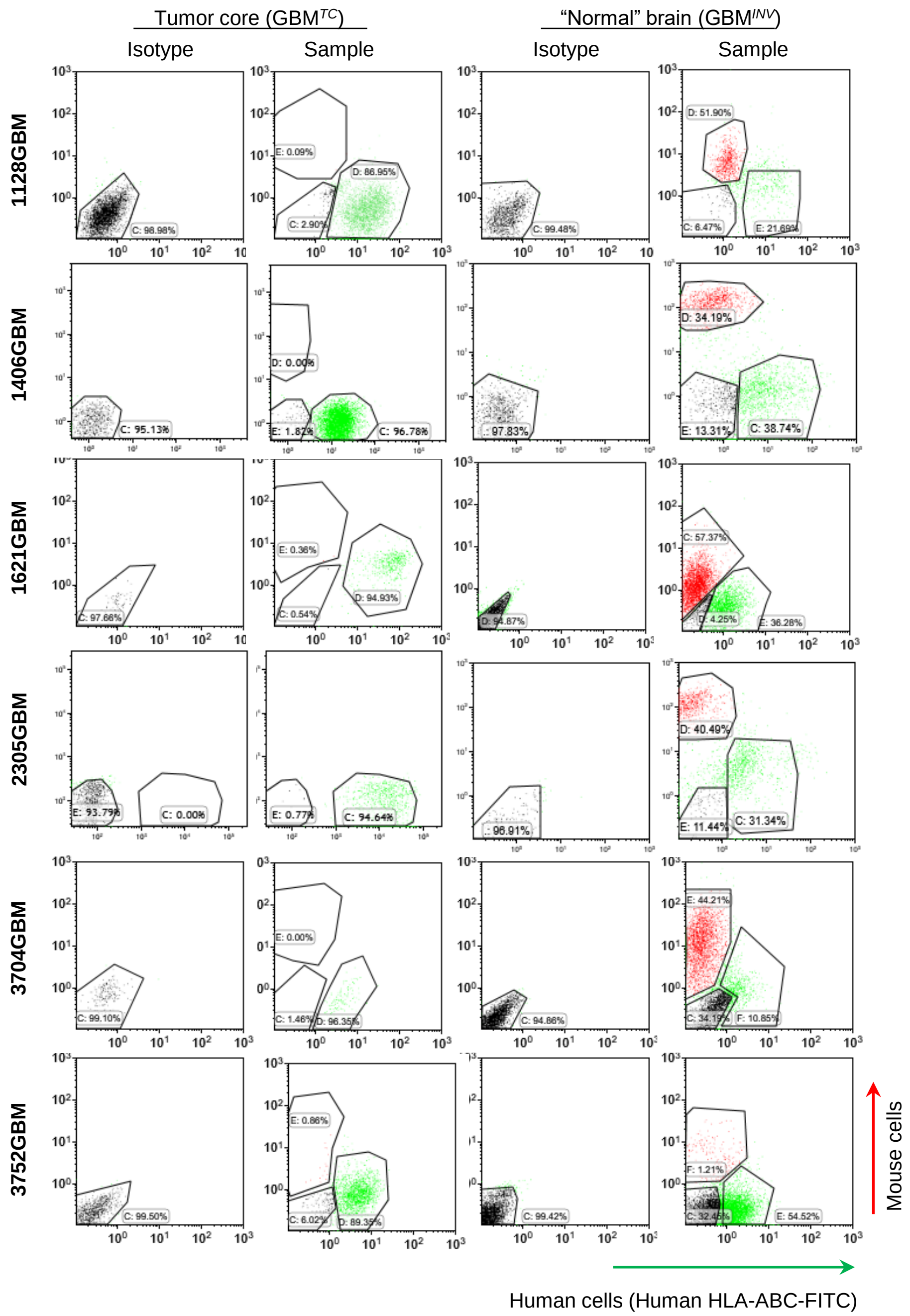

Suppl. Figure 2. FACS purification of GBM^{TC} cells from tumor core and GBM^{INV} cells from the matching "normal" mouse brains. Tumor core and "normal" mouse brains were dissociated into single cell suspensions using Gentle Dissociator (Miltenyi) and incubated with FITC-conjugated monoclonal antibodies against human HLA-ABC, -DR and APC-conjugated monoclonal antibodies against mouse major histocompatibility antigen. The mouse cells (APC-positive and FITC-negative) were gated out together with the dead cells (propidium iodine high).

a Down-regulating miRNA^{INV} in GBM^{INV} cells leads to decreased invasion *in vitro* without affecting proliferation

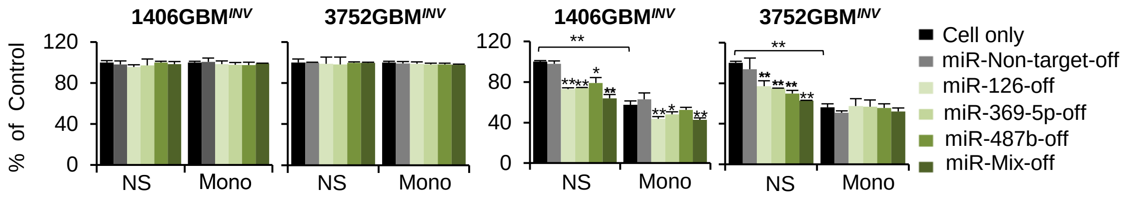

1406GBM^{INV}	NS						Mono					
p value	Cell only	miR-Non-target-off	miR-126-off	miR-369-5p-off	miR-487b-off	miR-Mix-off	Cell only	miR-Non-target-off	miR-126-off	miR-369-5p-off	miR-487b-off	miR-Mix-off
miR-Non-target-off	0.738		<0.001	<0.001	<0.001	<0.001	0.367		<0.001	<0.001	0.017	<0.001
miR-126-off	<0.001	<0.001		0.691	0.184	0.036	0.001	<0.001		0.279	0.048	0.757
miR-369-5p-off	<0.001	<0.001	0.691		0.407	0.033	0.035	<0.001	0.279		0.387	0.359
miR-487b-off	<0.001	<0.001	<0.001	0.407		<0.001	0.362	0.017	0.048	0.387		0.03
miR-Mix-off	<0.001	<0.001	<0.001	0.033	<0.001		<0.001	<0.001	0.757	0.359	0.03	

3752GBM^{INV}	NS						Mono					
p value	Cell only	miR-Non-target-off	miR-126-off	miR-369-5p-off	miR-487b-off	miR-Mix-off	Cell only	miR-Non-target-off	miR-126-off	miR-369-5p-off	miR-487b-off	miR-Mix-off
miR-Non-target-off	0.203		<0.001	<0.001	<0.001	<0.001	0.795		0.605	0.67	0.843	0.999
miR-126-off	<0.001	<0.001		0.522	0.17	0.002	0.995	0.605		0.905	0.999	0.828
miR-369-5p-off	<0.001	<0.001	0.522		0.245	0.009	0.995	0.67	0.905		0.998	0.842
miR-487b-off	<0.001	<0.001	0.17	0.245		0.201	0.982	0.843	0.999	0.998		0.949
miR-Mix-off	<0.001	<0.001	0.002	0.009	0.201		0.916	0.999	0.828	0.842	0.949	

b Up-regulating miRNA^{INV} in GBM^{TC} cells leads to increased invasion *in vitro* without affecting proliferation

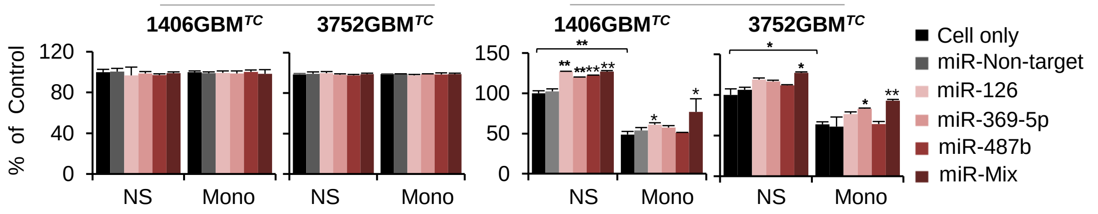

1406GBM^{TC}	NS						Mono					
miRNA	Cell only	miR-Non-target	miR-126	miR-369-5p	miR-487b	miR-Mix	Cell only	miR-Non-target	miR-126	miR-369-5p	miR-487b	miR-Mix
miR-Non-target	0.879		<0.001	<0.001	<0.001	<0.001	0.614		0.323	0.74	0.723	<0.001
miR-126	<0.001	<0.001		0.323	0.614	1	0.022	0.323		0.736	0.096	0.005
miR-369-5p	<0.001	<0.001	0.323		0.787	0.284	0.15	0.74	0.736		0.4	<0.001
miR-487b	<0.001	<0.001	0.614	0.787		0.533	0.506	0.723	0.096	0.4		<0.001
miR-Mix	<0.001	<0.001	1	0.284	0.533		<0.001	<0.001	0.005	<0.001	<0.001	

3752GBM^{TC}	NS						Mono					
p value	Cell only	miR-Non-target	miR-126	miR-369-5p	miR-487b	miR-Mix	Cell only	miR-Non-target	miR-126	miR-369-5p	miR-487b	miR-Mix
miR-Non-target	0.281		0.009	0.033	0.287	<0.001	0.686		0.003	<0.001	0.805	<0.001
miR-126	<0.001	0.009		0.489	0.195	0.124	0.007	0.003		0.246	0.007	<0.001
miR-369-5p	<0.001	0.033	0.489		0.297	0.034	<0.001	<0.001	0.246		<0.001	0.037
miR-487b	0.017	0.287	0.195	0.297		0.001	0.969	0.805	0.007	<0.001		<0.001
miR-Mix	<0.001	<0.001	0.124	0.034	0.001		<0.001	<0.001	<0.001	0.037	<0.001	

Suppl. Figure 3. *In vitro* functional analysis of miRNA^{INV} on cell proliferation and invasion in GBM^{TC} and GBM^{INV} cells. (**a**) Loss-of-function of miRNA^{INV} in the invasive GBM (GBM^{INV}) cells from two models IC-1406GBM (***1406GBM^{INV}***) and IC-3752GBM (***3752GBM^{INV}***). Cell proliferation and invasive capacity after silencing miR-126, -369-5p, and -487b with Lentivirus-miRNA-off. (**b**) Gain-of-function of miRNA^{INV} in the tumor core (GBM^{TC}) cells from two models (1406GBM^{TC}) and 3752GBM^{TC}). Cells were maintained both as neurosphere (***NS***) and monolayer (***Mono***), and all the data were normalized to the NS cell only group. In addition to the graphs (that were presented in Figure 4A), *P* values of pair-wise comparisons of tumor cell invasion were listed. (**$P < 0.01$, *$P < 0.05$ compared to the control group by two-way ANOVA, n = 3. Data are shown as mean ± SD).

a Down-regulation of miRNA^{TC} in GBM^{TC} cells leads to increased invasion *in vitro* without affecting proliferation

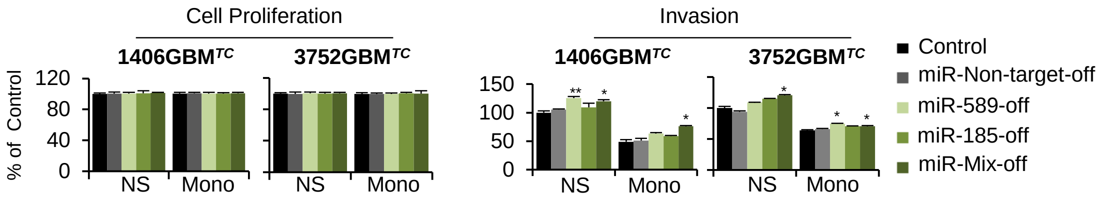

**1406GBM^{TC}** miRNA^{TC}	NS (*P value*)					Mono (*P value*)				
	Cell only	miR-Non-target-off	miR-589-off	miR-185-off	miR-Mix-off	Cell only	miR-Non-target-off	miR-589-off	miR-185-off	miR-Mix-off
miR-Non-target-off	0.67		0.007	0.684	0.019	0.67		0.113	0.339	<0.001
miR-589-off	<0.001	0.007		0.031	0.62	0.063	0.113		0.638	0.125
miR-185-off	0.288	0.684	0.031		0.078	0.211	0.339	0.638		0.025
miR-Mix-off	0.002	0.019	0.62	0.078		<0.001	<0.001	0.125	0.025	

**3752GBM^{TC}** miRNA^{TC}	NS (*P value*)					Mono (*P value*)				
	Cell only	miR-Non-target-off	miR-589-off	miR-185-off	miR-Mix-off	Cell only	miR-Non-target-off	miR-589-off	miR-185-off	miR-Mix-off
miR-Non-target-off	0.305		0.004	<0.001	<0.001	0.604		0.15	0.548	0.005
miR-589-off	0.089	0.004		0.155	0.036	0.066	0.15		0.452	0.364
miR-185-off	0.005	<0.001	0.155		0.271	0.39	0.548	0.452		0.068
miR-Mix-off	<0.001	<0.001	0.036	0.271		0.002	0.005	0.364	0.068	

b Up-regulation of miRNA^{TC} in GBM^{INV} cells leads to decreased invasion *in vitro* without affecting proliferation

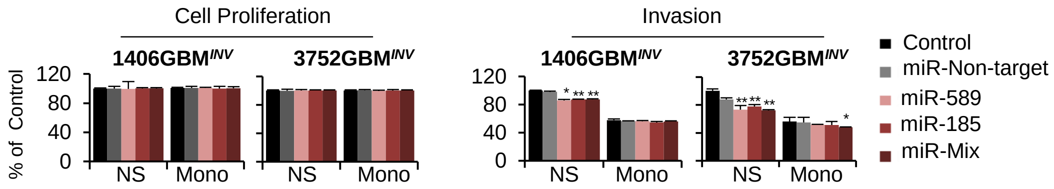

**1406GBM^{INV}** miRNA^{TC}	NS (*P value*)					Mono (*P value*)				
	Cell only	miR-Non-target	miR-589	miR-185	miR-Mix	Cell only	miR-Non-target	miR-589	miR-185	miR-Mix
miR-Non-target	0.746		<0.001	<0.001	<0.001	0.766		0.76	0.696	0.057
miR-589	<0.001	<0.001		0.598	0.803	0.439	0.76		0.822	0.391
miR-185	<0.001	<0.001	0.598		0.948	0.765	0.696	0.822		0.119
miR-Mix	<0.001	<0.001	0.803	0.948		0.014	0.057	0.391	0.119	

**3752GBM^{INV}** miRNA^{TC}	NS					Mono				
	Cell only	miR-Non-target	miR-589	miR-185	miR-Mix	Cell only	miR-Non-target	miR-589	miR-185	miR-Mix
miR-Non-target	<0.001		<0.001	<0.001	<0.001	0.65		0.264	0.168	0.005
miR-589	<0.001	<0.001		0.018	0.558	0.087	0.264		0.66	0.172
miR-185	<0.001	<0.001	0.018		0.007	0.037	0.168	0.66		0.244
miR-Mix	<0.001	<0.001	0.558	0.007		<0.001	0.005	0.172	0.244	

Suppl. Figure 4. *In vitro* functional analysis of miRNAs that were over-expressed in the tumor core cells (miRNA^{TC}) on cell proliferation and invasion in GBM^{TC} and GBM^{INV} cells. (**a**) Loss-of-function of miRNA^{TC}, miR-589 and miR-185, in tumor core GBM (GBM^{TC}) cells from two models IC-1406GBM

(***1406GBM***^{TC}) and IC-3752GBM (***3752GBM***^{TC}). Cell proliferation and invasive capacity after silencing miR-589, and -185 alone and in combination with Lentivirus-miRNA-off. (**b**) Gain-of-function of miRNA^{TC} in the invasive (GBM^{INV}) cells from two models (**1406GBM**^{INV} and **3752GBM**^{INV}) cells. Cells were maintained both as neurosphere (***NS***) and monolayer (***Mono***), and all the data were normalized to the NS cell only group. Both the graphs and *P* values of pair-wise comparisons of tumor cell invasion were listed. (**$P < 0.01$, *$P < 0.05$ compared to the control group by two-way ANOVA, n = 3. Data are shown as mean ± SD).

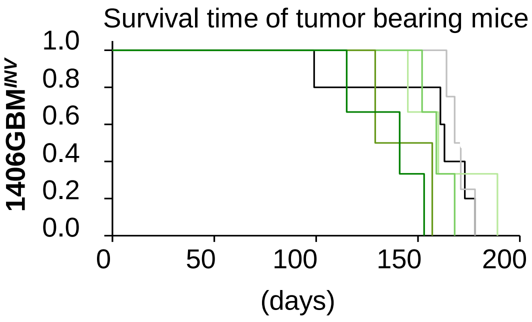

Group	Median Survival (days)
Cell only	155
miR-Non-target-off	164
miR-126-off	165
miR-369-5p-off	160
miR-487b-off	143
miR-Mix-off	141

Suppl. Figure 5. Log-rank analysis of survival time in tumor bearing mice. 1406GBM^{INV} cells were transduced with Lentivirus-miRNA-off to silence the miRNA^{INV}, followed by implantation into the brains of NOD/SCID mice (1x10^5 cells/mouse brain). Survival times of mice bearing confirmed intra-cerebral xenograft tumors were compared (P>0.05).

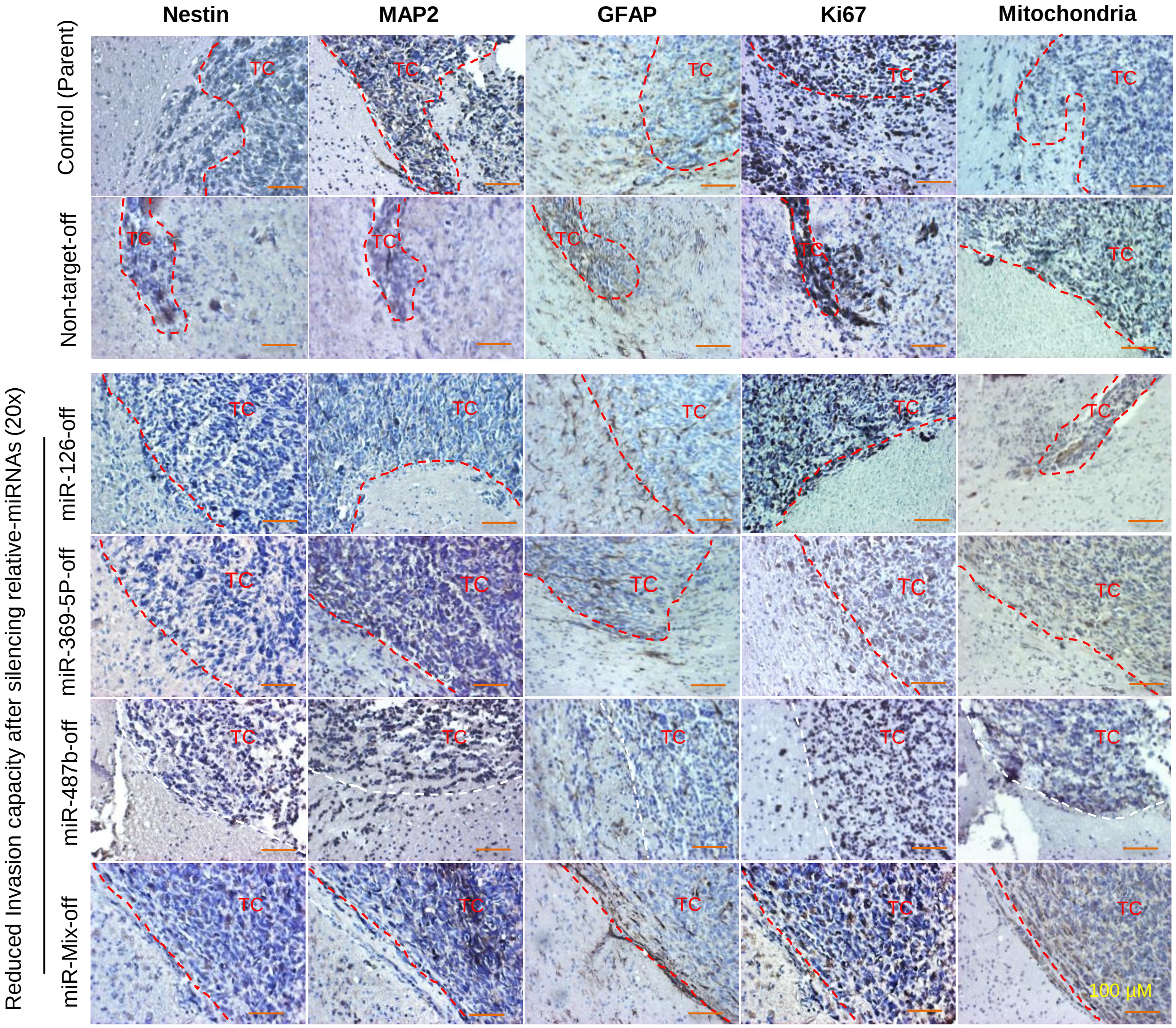

Summary of immunohistochemical staining after silencing miRNA^{INV} in IC-1406GBM

**1406GBM^{INV}**-Lenti-	Nestin	MAP2	GFAP	Ki67	Mitochondria
Control	++3*	+++2	+++2	+++4	+1
miR-Non-target-off	+2	++1	+++2	+++3	+++2
miR-126-off	-1	-1	+++1	+++3	+1
miR-369-5p-off	-1	+1	+++1	++3	+1
miR-487b-off	+2	+++3	+++1	+++4	+/-1
miR-Mix-off	-1	+2	+++1	+++3	+1

*Intensity of staining: weak(+); moderate(++); strong(+++). Percentage: 1=<25%, 2=25-50%, 3=50-75%, 4=>75%

Suppl. Figure 6. Changes of differentiation and proliferation markers in xenograft tumors derived from GBM^{INV} cells of IC-1406GBM following the silencing of miRNA^{INV}. Whole mouse brains were paraffin embedded and serially sectioned. Protein expression of stem cell marker (***Nestin***), neural

differentiation (***MAP2***), glial marker (***GFAP***), cell proliferation (***Ki67***) and mitochondria were examined through IHC (*top panel*). Borderlines of tumor core (***TC***) were highlighted (*dotted red lines*). Note the significantly reduced invasion in tumors of which miRNA[INV] (***miR-126-off, miR-369-5p-off, miR-487b-off, and the miR-mix-off***) were silenced. The intensity and relative abundance of positive IHC staining were summarized (*lower panel*). Scale bars represent 100 µM. Data are shown as mean ± SD.

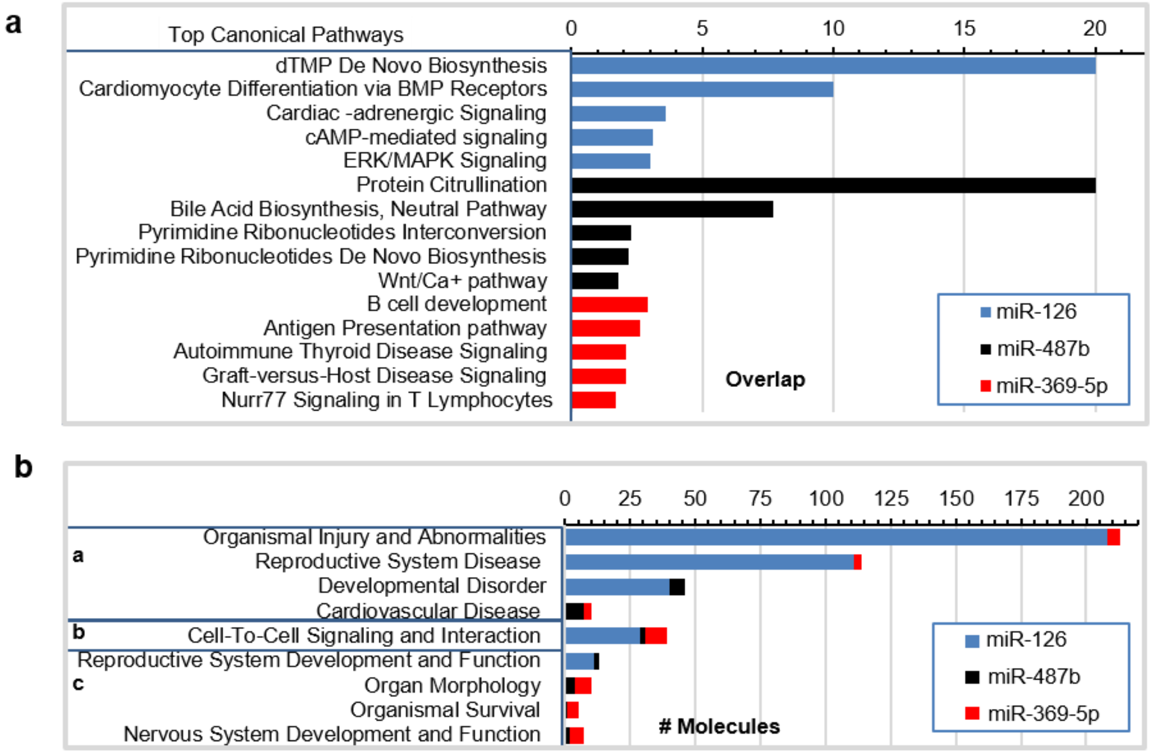

Suppl. Figure 7. The miRNA[INV] targeted a set of shared and private signaling pathways. **a and b** Pathway enrichment analysis and associated diseases of the differentially expressed target genes of was performed through Ingenuity with Fisher's exact test.

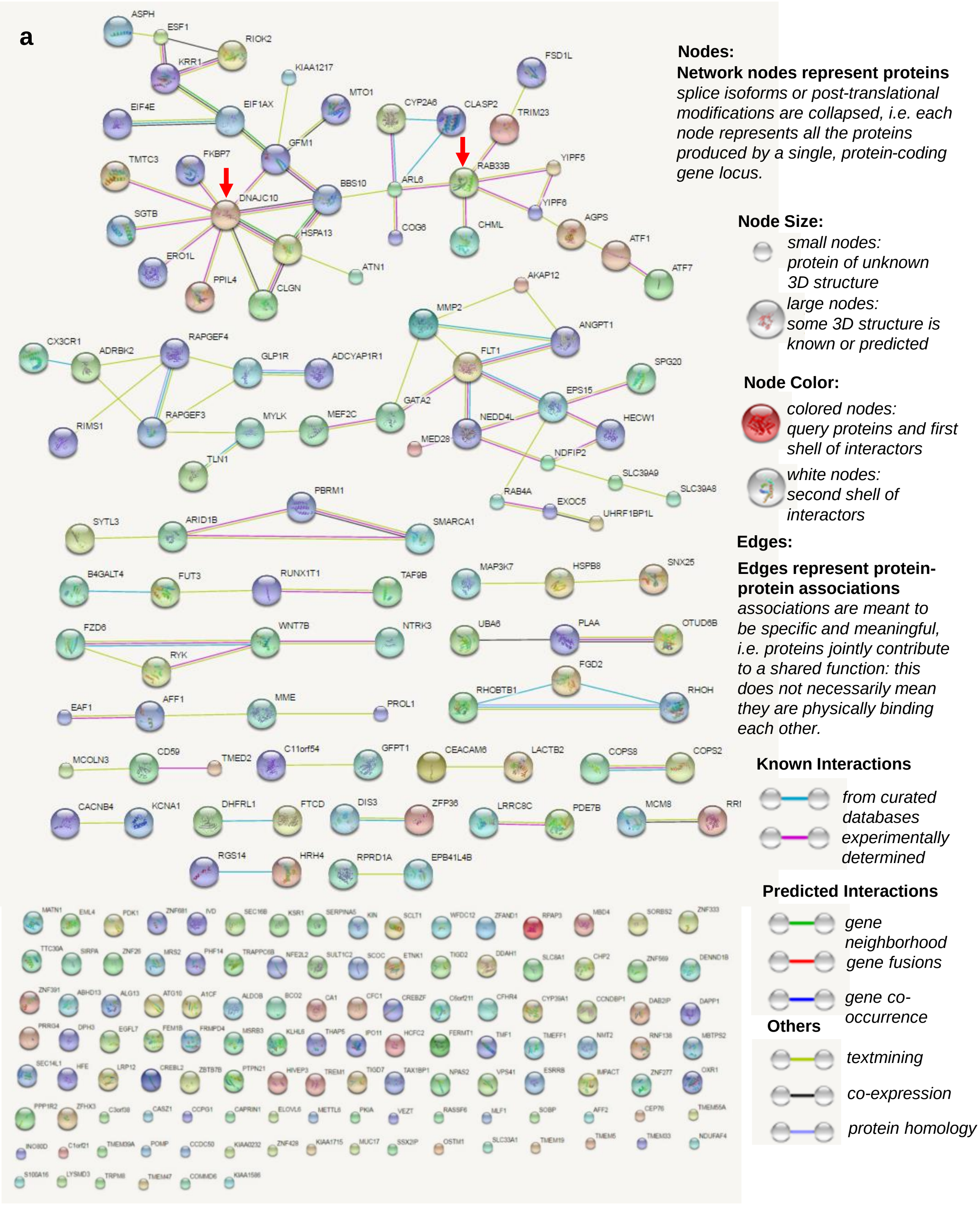
a
ASPH
ESF1
RIOK2
KRR1
KIAA1217
FSD1L
EIF4E
EIF1AX
MTO1
CYP2A6
CLASP2
TRIM23
GFM1
TMTC3
FKBP7
RAB33B
YIPF5
BBS10
ARL6
DNAJC10
YIPF6
SGTB
AGPS
COG6
CHML
HSPA13
ATF1
ERO1L
ATN1
ATF7
PPIL4
CLGN
AKAP12
MMP2
ANGPT1
CX3CR1
RAPGEF4
ADRBK2
GLP1R
ADCYAP1R1
FLT1
SPG20
GATA2
EPS15
RAPGEF3
MYLK
MEF2C
RIMS1
NEDD4L
HECW1
MED28
NDFIP2
TLN1
SLC39A9
PBRM1
RAB4A
SLC39A8
EXOC5
SYTL3
ARID1B
UHRF1BP1L
SMARCA1
B4GALT4
FUT3
RUNX1T1
TAF9B
MAP3K7
HSPB8
SNX25
FZD6
WNT7B
NTRK3
UBA6
PLAA
OTUD6B
RYK
FGD2
RHOBTB1
RHOH
EAF1
AFF1
MME
PROL1
MCOLN3
CD59
TMED2
C11orf54
GFPT1
CEACAM6
LACTB2
COPS8
COPS2
CACNB4
KCNA1
DHFRL1
FTCD
DIS3
ZFP36
LRRC8C
PDE7B
MCM8
RRI
RGS14
HRH4
RPRD1A
EPB41L4B
Nodes:
Network nodes represent proteins
splice isoforms or post-translational modifications are collapsed, i.e. each node represents all the proteins produced by a single, protein-coding gene locus.
Node Size:
small nodes: protein of unknown 3D structure
large nodes: some 3D structure is known or predicted
Node Color:
colored nodes: query proteins and first shell of interactors
white nodes: second shell of interactors
Edges:
Edges represent protein-protein associations
associations are meant to be specific and meaningful, i.e. proteins jointly contribute to a shared function: this does not necessarily mean they are physically binding each other.
Known Interactions
from curated databases
experimentally determined
Predicted Interactions
gene neighborhood
gene fusions
gene co-occurrence
Others
textmining
co-expression
protein homology

b

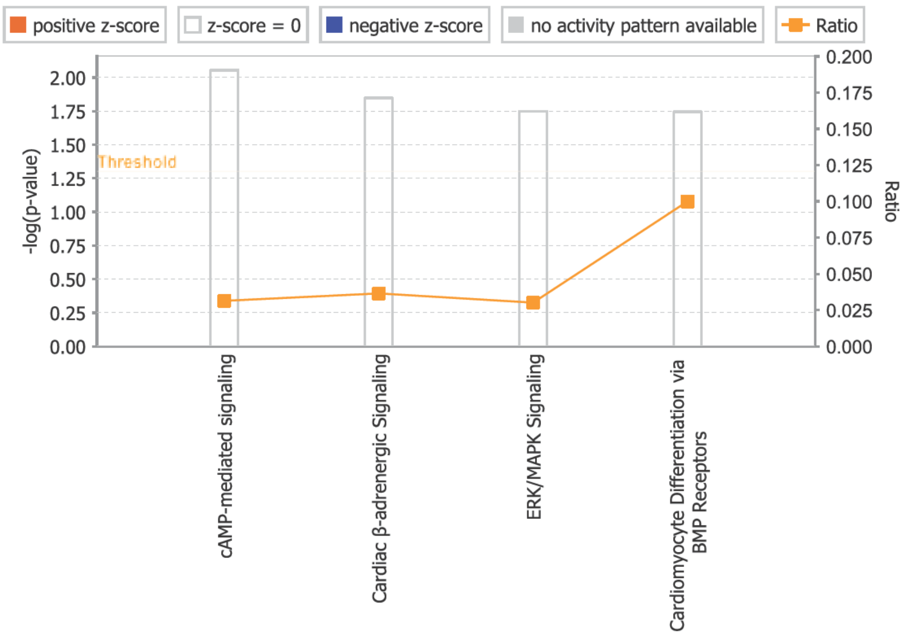

Suppl. Figure 8. Protein-protein interaction network and pathways targeted by miR-126 in GBM*INV* cells. (**a**) The target genes of miR-126 were identified through reverse revers search of TargetScan and their expression levels extracted from mRNA profiling of the same sets of GBM*INV* and GBM*TC* cells. The target genes of micro-RNA were inputted into STRING ((http://www.string-db.org) and 20 necessary proteins were added into the network except for the target genes. The output network is the protein-protein interactions between the targeted genes and those necessary added proteins. Red arrow indicated the node of genes. (**b**) Pathway enrichment analysis and associated diseases using genes from miR-126 targeted gene network. Fisher's exact test was employed. Significant enriched pathways with p<0.05 (equal to –log(p-value) larger than 1.301) were listed out. Ratio indicates the overlap rate of genes between the network genes and the pathway in Ingenuity database.

a

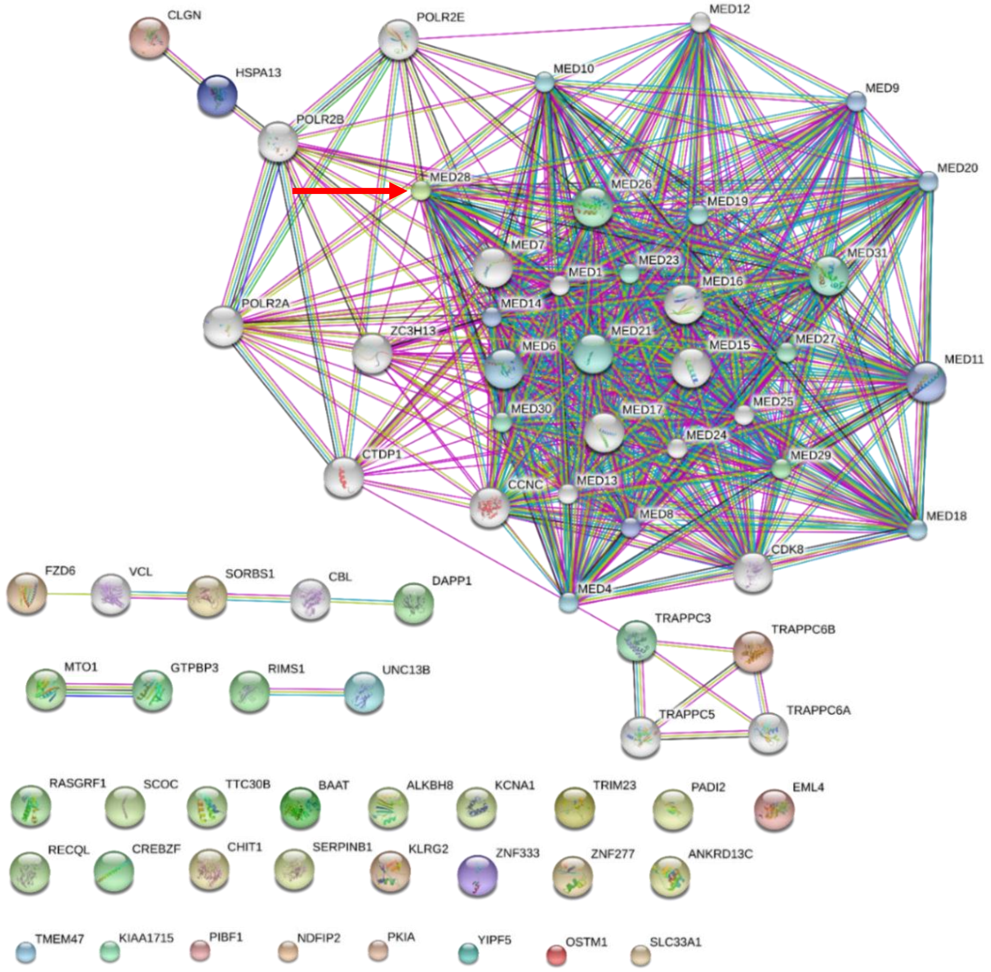
CLGN
HSPA13
POLR2E
MED12
MED10
MED9
POLR2B
MED20
MED28
MED26
MED19
MED7
MED1
MED23
MED31
MED16
MED14
POLR2A
ZC3H13
MED21
MED15
MED27
MED6
MED11
MED30
MED17
MED25
MED24
CTDP1
MED29
CCNC
MED13
MED8
MED18
CDK8
MED4
FZD6
VCL
SORBS1
CBL
DAPP1
TRAPPC3
TRAPPC6B
MTO1
GTPBP3
RIMS1
UNC13B
TRAPPC5
TRAPPC6A
RASGRF1
SCOC
TTC30B
BAAT
ALKBH8
KCNA1
TRIM23
PADI2
EML4
RECQL
CREBZF
CHIT1
SERPINB1
KLRG2
ZNF333
ZNF277
ANKRD13C
TMEM47
KIAA1715
PIBF1
NDFIP2
PKIA
YIPF5
OSTM1
SLC33A1

b

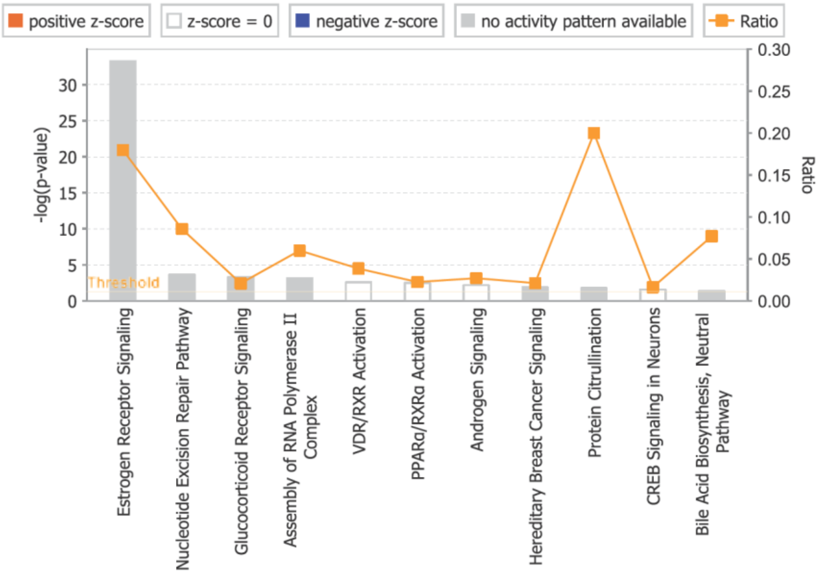
positive z-score
z-score = 0
negative z-score
no activity pattern available
Ratio
-log(p-value)
Ratio
0
5
10
15
20
25
30
0.00
0.05
0.10
0.15
0.20
0.25
0.30
Threshold
Estrogen Receptor Signaling
Nucleotide Excision Repair Pathway
Glucocorticoid Receptor Signaling
Assembly of RNA Polymerase II Complex
VDR/RXR Activation
PPARα/RXRα Activation
Androgen Signaling
Hereditary Breast Cancer Signaling
Protein Citrullination
CREB Signaling in Neurons
Bile Acid Biosynthesis, Neutral Pathway

Suppl. Figure 9. Protein-protein interaction network and pathways targeted by miR-487b in GBM^{INV} cells. (**a**) The target genes of miR-487b were identified through reverse revers search of TargetScan and their expression levels extracted from mRNA profiling of the same sets of GBM^{INV} and GBM^{TC} cells. The target genes of micro-RNA were inputted into STRING ((http://www.string-db.org) and 20 necessary proteins were added into the network except for the target genes. The output network is the protein-protein interactions between the targeted genes and those necessary added proteins. Red arrow indicated the node of genes. (**b**) Pathway enrichment analysis and associated diseases using genes from miR-487b targeted gene network. Fisher's exact test was employed. Significant enriched pathways with $p<0.05$ (equal to –log(p-value) larger than 1.301) were listed out. Ratio indicates the overlap rate of genes between the network genes and the pathway in Ingenuity database.

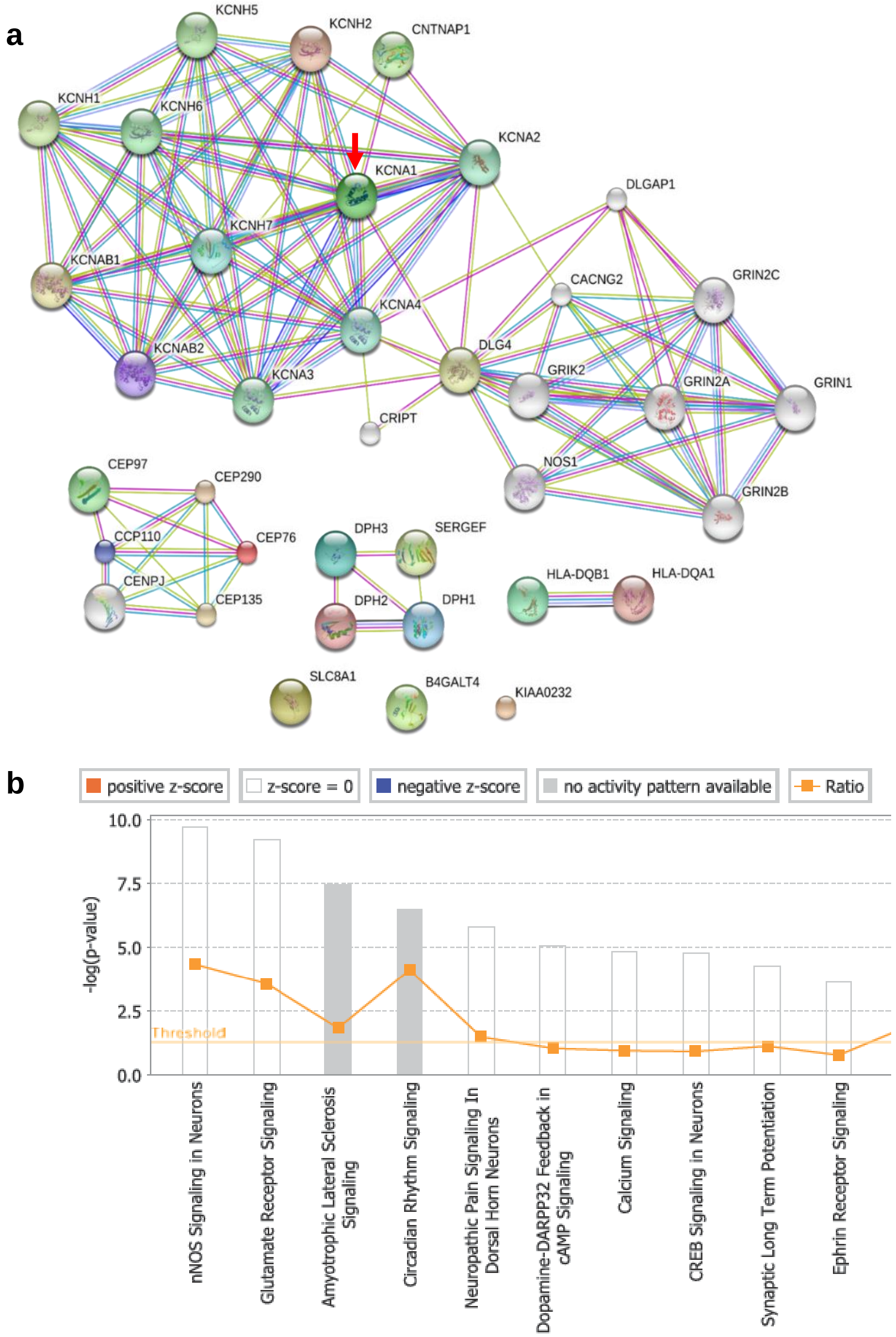

Suppl. Figure 10. Protein-protein interaction network and pathways targeted by miR-369-5p in GBM*INV* cells. (**a**) The target genes of miR-369-5p were identified through reverse revers search of TargetScan and their expression levels extracted from mRNA profiling of the same sets of GBM^{INV} and GBM^{TC} cells. The target genes of micro-RNA were inputted into STRING ((http://www.string-db.org)

and 20 necessary proteins were added into the network except for the target genes. The output network is the protein-protein interactions between the targeted genes and those necessary added proteins. Red arrow indicated the node of genes. (**b**) Pathway enrichment analysis and associated diseases using genes from miR-369-5p targeted gene network. Fisher's exact test was employed. Significant enriched pathways with $p<0.05$ (equal to –log(p-value) larger than 1.301) were listed out. Ratio indicates the overlap rate of genes between the network genes and the pathway in Ingenuity database.

a

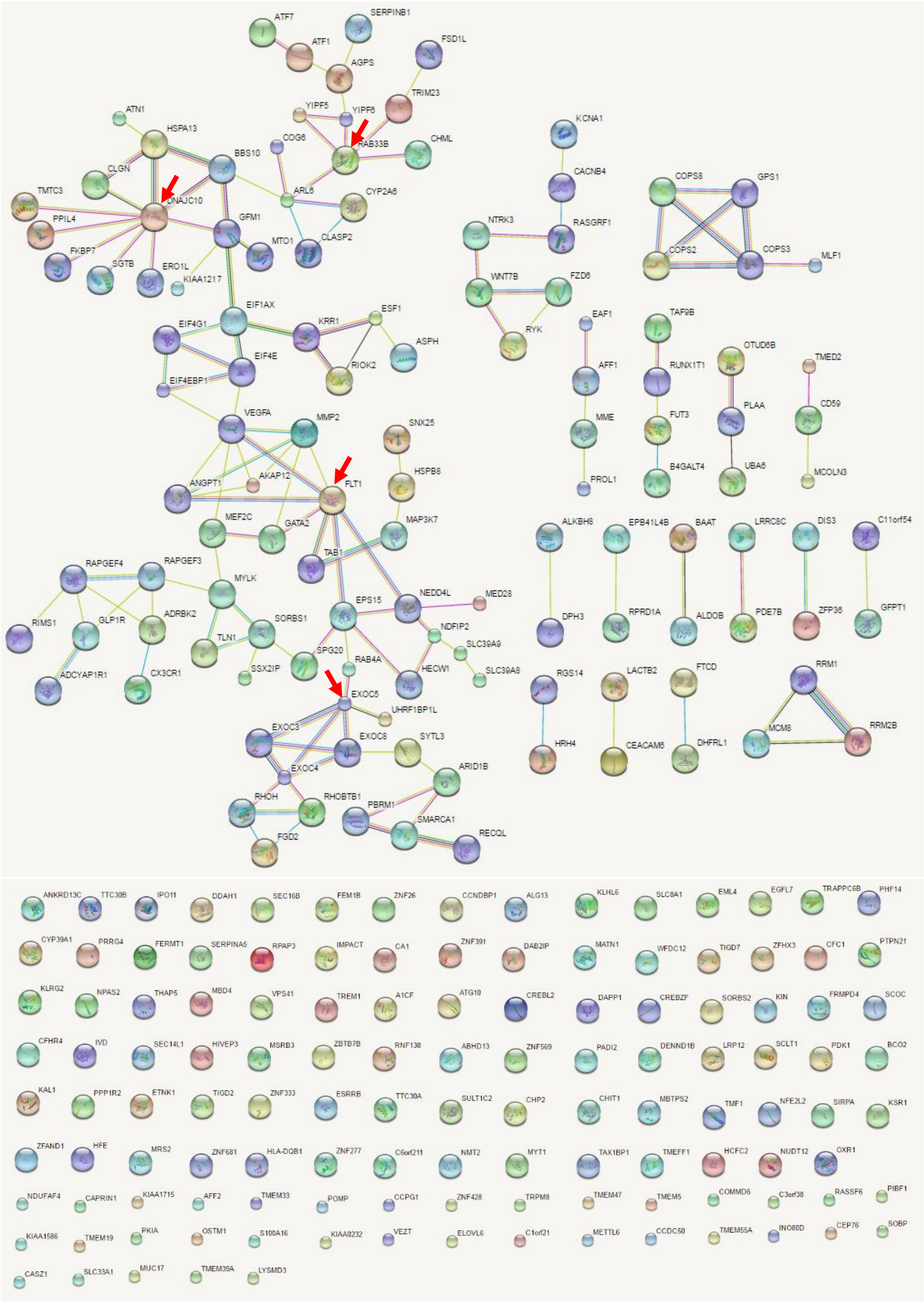
ATF7
SERPINB1
ATF1
FSD1L
AGPS
TRIM23
YIPF5
YIPF6
ATN1
HSPA13
COG6
RAB33B
CHML
BBS10
CLGN
ARL6
CYP2A6
TMTC3
DNAJC10
PPIL4
GFM1
CLASP2
MTO1
FKBP7
SGTB
ERO1L
KIAA1217
EIF1AX
ESF1
EIF4G1
KRR1
ASPH
EIF4E
RIOK2
EIF4EBP1
VEGFA
MMP2
SNX25
AKAP12
HSPB8
ANGPT1
FLT1
MEF2C
GATA2
MAP3K7
TAB1
RAPGEF4
RAPGEF3
MYLK
NEDD4L
MED28
ADRBK2
SORBS1
EPS15
RIMS1
GLP1R
TLN1
NDFIP2
SLC39A9
SPG20
RAB4A
SSX2IP
HECW1
SLC39A8
ADCYAP1R1
CX3CR1
EXOC5
UHRF1BP1L
EXOC3
EXOC8
SYTL3
EXOC4
ARID1B
RHOH
RHOBTB1
PBRM1
SMARCA1
FGD2
RECQL
KCNA1
CACNB4
RASGRF1
NTRK3
WNT7B
FZD6
RYK
COPS8
GPS1
COPS2
COPS3
MLF1
EAF1
AFF1
MME
PROL1
TAF9B
RUNX1T1
FUT3
B4GALT4
OTUD6B
PLAA
UBA6
TMED2
CD59
MCOLN3
ALKBH8
DPH3
EPB41L4B
RPRD1A
BAAT
ALDOB
LRRC8C
PDE7B
DIS3
ZFP36
C11orf54
GFPT1
RGS14
HRH4
LACTB2
CEACAM8
FTCD
DHFRL1
RRM1
MCM8
RRM2B
ANKRD13C
TTC30B
IPO11
DDAH1
SEC16B
FEM1B
ZNF26
CCNDBP1
ALG13
KLHL6
SLC8A1
EML4
EGFL7
TRAPPC6B
PHF14
CYP39A1
PRRG4
FERMT1
SERPINA6
RPAP3
IMPACT
CA1
ZNF391
DAB2IP
MATN1
WFDC12
TIGD7
ZFHX3
CFC1
PTPN21
KLRG2
NPAS2
THAP5
MBD4
VPS41
TREM1
A1CF
ATG10
CREBL2
DAPP1
CREBZF
SORBS2
KIN
FRMPD4
SCOC
CFHR4
IVD
SEC14L1
HIVEP3
MSRB3
ZBTB7B
RNF138
ABHD13
ZNF569
PADI2
DENND1B
LRP12
SCLT1
PDK1
BCO2
KAL1
PPP1R2
ETNK1
TIGD2
ZNF333
ESRRB
TTC30A
SULT1C2
CHP2
CHIT1
MBTPS2
TMF1
NFE2L2
SIRPA
KSR1
ZFAND1
HFE
MRS2
ZNF681
HLA-DQB1
ZNF277
C6orf211
NMT2
MYT1
TAX1BP1
TMEFF1
HCFC2
NUDT12
OXR1
NDUFAF4
CAPRIN1
KIAA1715
AFF2
TMEM33
POMP
CCPG1
ZNF428
TRPM8
TMEM47
TMEM5
COMMD6
C3orf38
RASSF6
PIBF1
KIAA1586
TMEM19
PKIA
OSTM1
S100A16
KIAA0232
VEZT
ELOVL6
C1orf21
METTL6
CCDC50
TMEM55A
INO80D
CEP76
SOBP
CASZ1
SLC33A1
MUC17
TMEM39A
LYSMD3

b

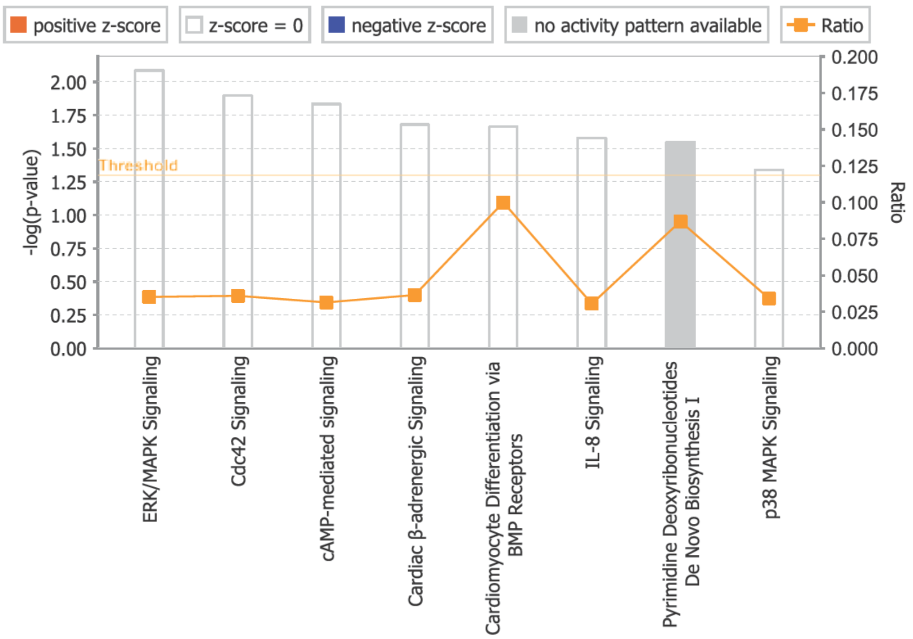

Suppl. Figure 11. Protein-protein interaction network and pathways targeted by the three miRNAs^{INV} in GBM^{INV} cells. (**a**) All the differentially expressed target genes of the three miRNAs were combined together and analyzed through STRING. Red arrow indicated the node of genes. (**b**) Pathway enrichment analysis and associated diseases using genes from all the three miRNA^{INV} targeted gene network. Fisher's exact test was employed. Significant enriched pathways with p<0.05 (equal to –log(p-value) larger than 1.301) were listed out. Ratio indicates the overlap rate of genes between the network genes and the pathway in Ingenuity database.

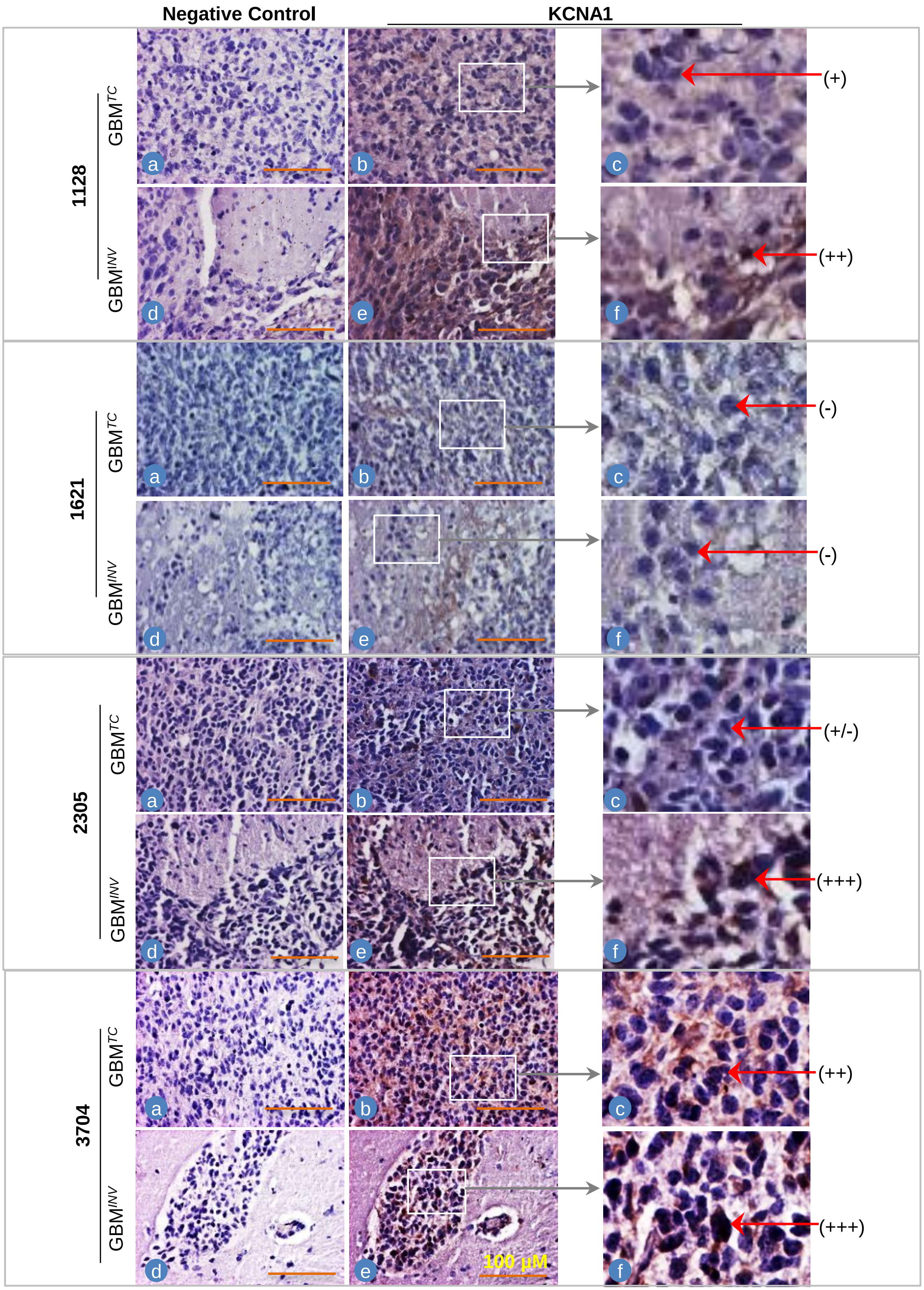

Negative Control
KCNA1
1128
GBM^TC
GBM^INV
(+)
(++)
1621
(-)
(-)
2305
(+/-)
(+++)
3704
(++)
(+++)
100 μM
a
b
c
d
e
f

Suppl. Figure 12. *KCNA1* protein expression was enriched in the invasive cells *in vivo* in PDOX mouse models of pGBM. KCNA1 protein expression in the invasive front (GBM^{INV}) and tumor core (GBM^{TC}) examined through IHC in the whole mouse sections of the four pGBM PDOX models (IC-1128GBM, IC-1621GBM, IC-2305GBM and IC-3704GBM) (IC-1406GBM and IC-3752GBM were shown in Fig. 5E) (40x). Slides incubated without primary antibodies were included as negative control.

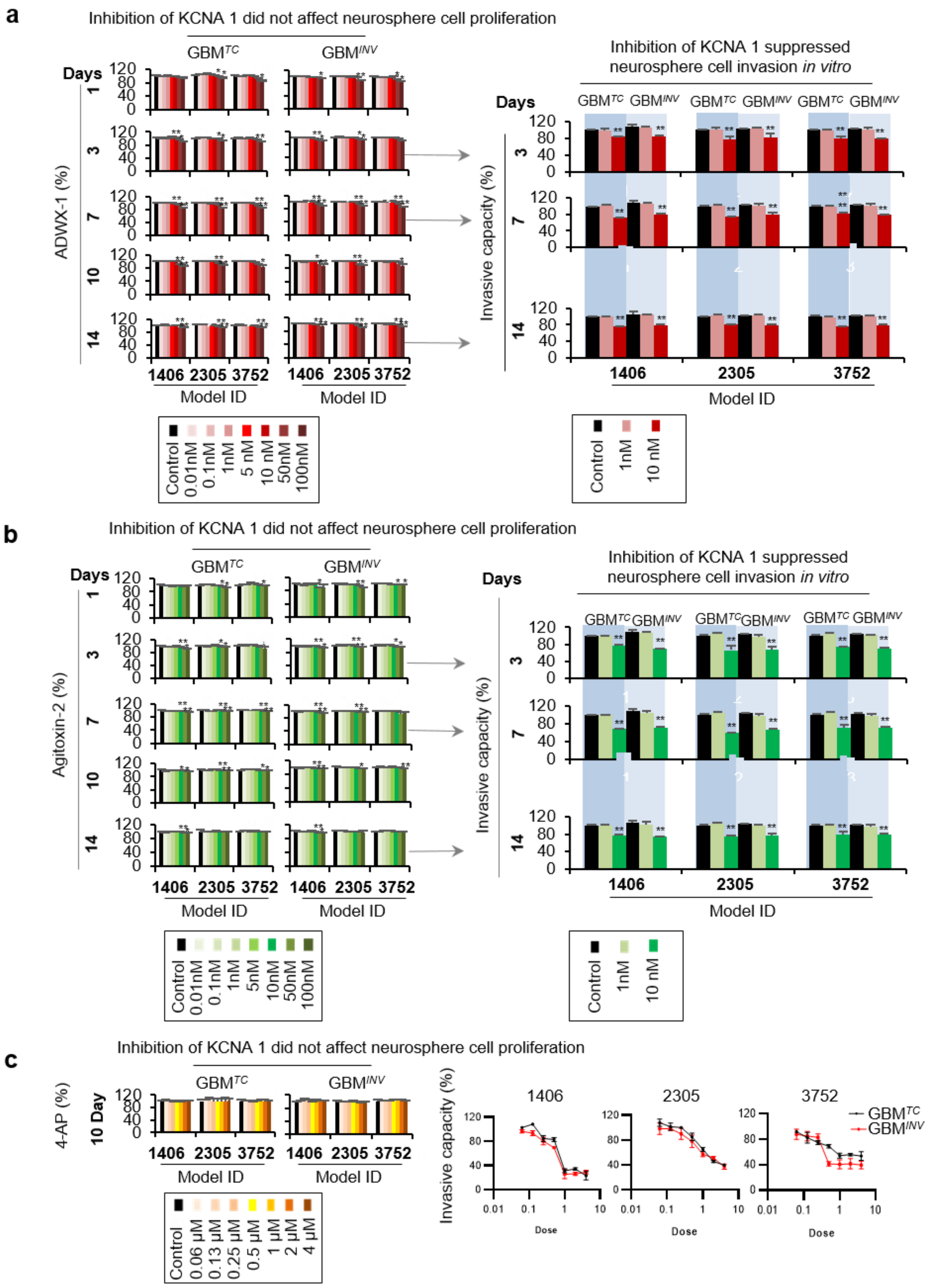
a
Inhibition of KCNA 1 did not affect neurosphere cell proliferation
GBM^TC
GBM^INV
Days
1
3
7
10
14
ADWX-1 (%)
120
80
40
0
1406 2305 3752
Model ID
Control
0.01nM
0.1nM
1nM
5 nM
10 nM
50nM
100nM
Inhibition of KCNA 1 suppressed neurosphere cell invasion in vitro
Invasive capacity (%)
1406
2305
3752
Control
1nM
10 nM
b
Agitoxin-2 (%)
Control
0.01nM
0.1nM
1nM
5nM
10nM
50nM
100nM
c
4-AP (%)
10 Day
Control
0.06 µM
0.13 µM
0.25 µM
0.5 µM
1 µM
2 µM
4 µM
Dose
0.01
0.1
1
10
GBM^TC
GBM^INV

Suppl. Figure 13. Pharmacological inhibition of *KCNA1* suppresses pGBM invasion *in vitro.* Impact of *KCNA1* inhibitor ADWX-1 (**a**), Agitoxin-2 (**b**) and 4-AP (**c**) on cell proliferation were examined in three pairs of neurosphere cultures of GBM^{TC} and GBM^{INV} cells derived from three IC-1406GBM (***1406***), IC-2305GBM (***2305***) and IC-3752GBM (***3752***) models. Tumor cells were exposed to ADWX-1 and Agitoxin-2 at 0.01-100 nM for 1-14 days and 0.06-4 µM for 7 and 10 days for 4-AP. Changes of cell proliferation were examined with CCK-8 kit assay (*$P < 0.05$) (*left panels* of A-C); and the suppression of cell invasion was examined by treating neurospheres derived from tumor core (GBM^{TC}) and invasive (GBM^{INV}) cells for 3, 7 and 14 days for ADWX-1 and Agitoxin-2 and 7 and 10 days for 4-AP. Changes of cell invasion was examined with the CytoSelect 24-Well Cell Invasion Assay. Data are shown as mean ± SD. *$P < 0.05$.
